# Design of a mutation-integrated trimeric RBD with broad protection against SARS-CoV-2

**DOI:** 10.1038/s41421-022-00383-5

**Published:** 2022-02-15

**Authors:** Yu Liang, Jing Zhang, Run Yu Yuan, Mei Yu Wang, Peng He, Ji Guo Su, Zi Bo Han, Yu Qin Jin, Jun Wei Hou, Hao Zhang, Xue Feng Zhang, Shuai Shao, Ya Nan Hou, Zhao Ming Liu, Li Fang Du, Fu Jie Shen, Wei Min Zhou, Ke Xu, Ru Qin Gao, Fang Tang, Ze Hua Lei, Shuo Liu, Wei Zhen, Jin Juan Wu, Xiang Zheng, Ning Liu, Shi Chen, Zhi Jing Ma, Fan Zheng, Si Yu Ren, Zhong Yu Hu, Wei Jin Huang, Gui Zhen Wu, Chang Wen Ke, Qi Ming Li

**Affiliations:** 1 The Sixth Laboratory, National Vaccine and Serum Institute (NVSI), Beijing, China; 2 National Engineering Center for New Vaccine Research, Beijing, China; 3 Guangdong Provincial Institute of Public Health, Guangdong Provincial Center for Disease Control and Prevention, Guangzhou, Guangdong China; 4 National Institute for Food and Drug Control (NIFDC), Beijing, China; 5 National Institute for Viral Disease Control and Prevention, Chinese Center for Disease Control and Prevention (China CDC), Beijing, China; 6 Qingdao Centers for Disease Control and Prevention, Qingdao, Shandong China

**Keywords:** Immunology, Biological techniques

## Abstract

The continuous emergence of SARS-CoV-2 variants highlights the need of developing vaccines with broad protection. Here, according to the immune-escape capability and evolutionary convergence, the representative SARS-CoV-2 strains carrying the hotspot mutations were selected. Then, guided by structural and computational analyses, we present a mutation-integrated trimeric form of spike receptor-binding domain (mutI-tri-RBD) as a broadly protective vaccine candidate, which combined heterologous RBDs from different representative strains into a hybrid immunogen and integrated immune-escape hotspots into a single antigen. When compared with a homo-tri-RBD vaccine candidate in the stage of phase II trial, of which all three RBDs are derived from the SARS-CoV-2 prototype strain, mutI-tri-RBD induced significantly higher neutralizing antibody titers against the Delta and Beta variants, and maintained a similar immune response against the prototype strain. Pseudo-virus neutralization assay demonstrated that mutI-tri-RBD also induced broadly strong neutralizing activities against all tested 23 SARS-CoV-2 variants. The in vivo protective capability of mutI-tri-RBD was further validated in hACE2-transgenic mice challenged by the live virus, and the results showed that mutI-tri-RBD provided potent protection not only against the SARS-CoV-2 prototype strain but also against the Delta and Beta variants.

## Introduction

Severe acute respiratory syndrome coronavirus 2 (SARS-CoV-2) has spread rapidly around the world since the end of 2019 and resulted in a pandemic of this new coronavirus infections^1,2^, which has imposed a big threat to public health and a heavy burden on the global economy. To date, significant achievement has been made in the coronavirus disease 2019 (COVID-19) vaccine development and several vaccines are authorized by World Health Organization (WHO) for emergency use^3–5^. However, new variants are continuously emerging due to the fast evolution of the virus. As of October 2021, four SARS-CoV-2 strains, including Alpha (B.1.1.7), Beta (B.1.351), Gamma (P.1), and Delta (B.1.617.2), are classified as variants of concern by WHO. More lines of evidence have demonstrated the increased transmissibility and virulence of these viriants^6–8^. These mutations may also potentially lead to immune escape from the host defense system^9^. The Beta and Gamma variants have been proved to be able to evade the neutralizing antibodies elicited by natural infections and vaccinations^10^. The ChAdOx1 nCoV-19 vaccine has been proved in clinical trials to be unprotective against the mild-to-moderate COVID-19 caused by the Beta variant^11^. The emergence of SARS-CoV-2 variants within the lineage B.1.617, including Kappa (B.1.617.1), Delta (B.1.617.2), and B.1.617.3, has led to a new wave of infections, and several preliminary studies suggested that the B.1.617 variants were less sensitive to the sera from vaccinated individuals and resistant to some monoclonal antibodies^12,13^. Exhaustive analysis of the available SARS-CoV-2 sequences has revealed that several immune-resistant mutations in the circulating variants were also evolutionarily convergent hotspots^14^, indicating the possible recurrence of these mutations individually or combinedly in the future. The possible immune escape of the emerging SARS-CoV-2 variants has raised concerns on the efficacy of existing vaccines and raised urgent needs of developing vaccines with broad-spectrum protection against variants evolved.

The conventional method for the development of a multi-protective vaccine is to produce multivalent vaccines through a mixture of strain-specific monovalent vaccines together. But this strategy is time-consuming and cost-expensive. Another effective method to develop multivalent vaccine is the construction of chimeric vaccine via integrating multiple circulating variants into a single antigen by structure- and computation-based rational design. The commonly used strategy in chimeric protein design is the replacement of structurally homologous modular domains without changing the overall structure of the antigenic protein, e.g., a chimeric Sarbecovirus spike protein constructed by Martinez et al.^15^. However, for the highly variable virus, another more promising strategy for broadly protective vaccine design is to integrate rather than substitute immunodominant domains into a single immunogen.

The spike (S) glycoproteins on SARS-CoV-2 surface play a key role in recognition of the host cell receptor, i.e., human angiotensin-converting enzyme 2 (hACE2), to mediate virus entry into the cell. S protein is naturally self-assembled into a homotrimer anchored onto the viral membrane. Each monomer of S protein is composed of two subunits, S1 and S2^16,17^. The receptor-binding domain (RBD) of S1 subunit protrudes from the viral surface and directly interacts with the receptor hACE2. Immunological studies showed that S1 RBD contains multiple neutralizing epitopes, which are immunodominant in eliciting neutralizing antibody responses against SARS-CoV-2 infection^18–20^. In addition, S1 RBD-based vaccine candidates have been shown to elicit neutralizing antibody responses in vitro against both the pseudo and live SARS-CoV-2, and induce protective immunity in vivo in non-human primates^21,22^. However, the RBD monomer has limitations in immunogenicity for vaccine development due to its small molecular size^23,24^. To improve the immunogenicity of RBD, polymerization would be a potentially effective strategy by increasing its molecular size to develop the immunogen with multiple copies of antigenic determinants. Given that native S protein is self-assembled into a trimer and S1 RBDs naturally exist in a homo-trimeric form, it is reasonably speculated that a trimeric form of RBD resembling its native conformational arrangements could achieve better immunogenic properties. Structural analysis of S trimer indicates that the N- and C-terminus of RBD are close to each other and there exists a long loop with high flexibility at both termini, which potentially allow the formation of RBD trimer (tri-RBD) through end-to-end connection to mimic the RBD arrangements in the natural S protein trimer. Furthermore, the tri-RBD that accommodates three RBDs in one molecule enables us to construct a multivalent vaccine candidate, in which three RBDs are individually derived from different circulating SARS-CoV-2 variants and co-assembled into one hybrid trimer.

In the present study, according to the immune-escape capability and evolutionary convergence, the representative SARS-CoV-2 circulating strains that harbor the hotspot mutations were selected. Then, guided by structural and computational analyses, we designed a mutation-integrated trimeric form of RBD, named mutI-tri-RBD, as a vaccine candidate with broadly protective capability, in which the RBDs from the representative SARS-CoV-2 strains were connected end-to-end and co-assembled to possibly mimic the native trimeric arrangements in the natural S protein trimer. It is worth pointing out that a homo-tri-RBD vaccine candidate developed based on SARS-CoV-2 prototype strain by our group is currently in phase II clinical trial (NCT04869592). Preliminary results of the trial demonstrated that homo-tri-RBD is safe and well tolerated. The neutralizing antibody geometric mean titers (GMTs) after three vaccine doses in the participants were about 3–5-fold higher than those of human convalescent sera (data not yet published). In the present study, the homo-tri-RBD was used as a comparative control. Then, the biochemical characterizations and immunological properties of the recombinant mutI-tri-RBD were evaluated comprehensively and compared with those of homo-tri-RBD, which support the mutI-tri-RBD as a promising immunogen for further clinical developments.

## Results

### Structure and computation-guided construction of the mutI-tri-RBD as an immunogen

A hybrid trimeric RBD named mutI-tri-RBD was constructed by incorporating multiple immunodominant RBDs from different circulating variants into a single immunogenic molecule. The rationale for the design of mutI-tri-RBD is as follows: (1) In the native S protein, the N- and C-terminus of the RBD are close to each other, which enables multimerization of RBDs through end-to-end connections without serious steric clashes. (2) There exist long flexible loops at both termini of the RBD, which would facilitate the connection of these different RBDs without destroying the core structure of the individual domain. (3) The tri-RBD construction strategy enables the realization of multivalent vaccine via a hybrid connection of RBDs from different circulating strains of SARS-CoV-2 into a single immunogenic molecule.

**Fig. 1:**
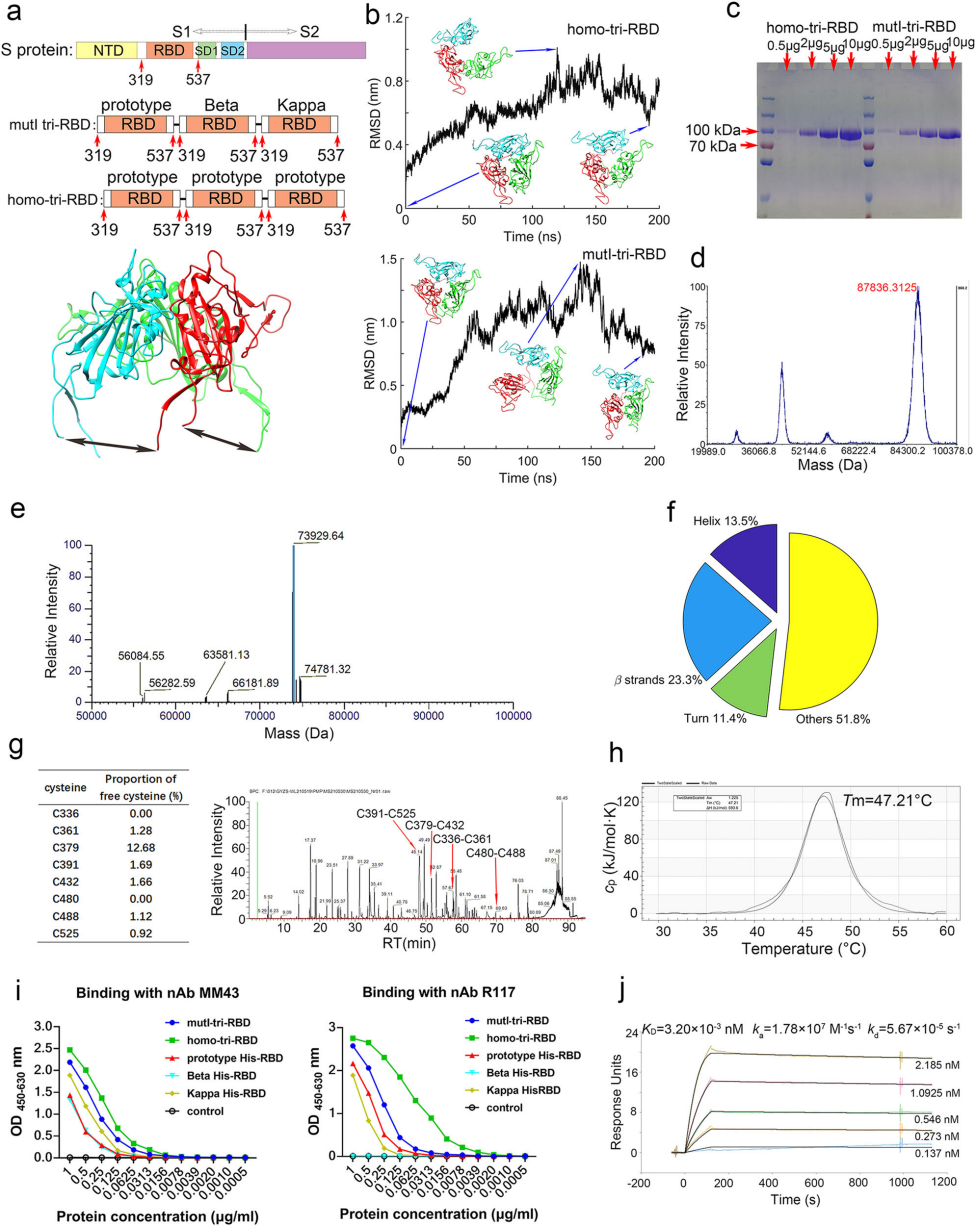
Structure-guided design, production, and characterization of the mutI-tri-RBD. **a** A schematic illustration of the mutI-tri-RBD and homo-tri-RBD design schemes. The RBD region comprising the residues 319–537 was truncated from the S protein, and three truncated RBDs were connected end-by-end to construct the trimeric forms of RBD. In mutI-tri-RBD, three RBDs were individually derived from three different circulating SARS-CoV-2 strains, i.e., the prototype, Beta and Kappa. In homo-tri-RBD, the three RBD units were all truncated from the prototype strain. In the upper subfigure, the S1 and S2 subunits of the S protein, as well as NTD, RBD, SD1, and SD2 in the S1 subunit, are marked. The lower subfigure displays the natural trimeric arrangement of RBDs in the native structure of S trimer. The arrows indicate the direct connections of the N- and C- terminals between different RBDs. **b** Structural modeling and MD simulation of the designed homo-tri-RBD (upper subfigure) and mutI-tri-RBD (lower subfigure). Time-evolution of the C_α_ root-mean square deviation (RMSD) of the modeled structure during MD simulation, as well as several snapshot conformations in the simulation, is displayed. **c** SDS-PAGE profiles of increasing amounts of the recombinant mutI-tri-RBD and homo-tri-RBD proteins expressed by HEK293T cells. **d** Molecular weight of mutI-tri-RBD determined by MALDI-TOF MS. **e** Molecular weight of mutI-tri-RBD after deglycosylation determined by UPLC-MS. **f** Secondary structure contents of mutI-tri-RBD protein analyzed by circular dichroism spectrometry. **g** Left: the proportions of free sulfhydryl for all the cysteine residues in mutI-tri-RBD. Right: the disulfide linkages in the recombinant mutI-tri-RBD protein detected by liquid chromatography-mass spectrometry. Only the disulfide bonds in one RBD unit are listed. **h** Differential scanning calorimetry thermograms of the recombinant mutI-tri-RBD protein. **i** The binding capability of the designed mutI-tri-RBD and homo-tri-RBD proteins with two anti-RBD monoclonal nAbs, i.e., MM43 and R117, evaluated by ELISA. As controls, the binding activities with the monoclonal nAbs for the monomeric his-tagged RBDs from the prototype, Beta, and Kappa SARS-CoV-2 strains were also measured. **j** The binding profiles of the recombinant mutI-tri-RBD with hACE2 detected by surface plasmon resonance assay.

From the native tertiary structure of S protein, a RBD truncation scheme that meets the above considerations was designed, as shown in Supplementary Fig. S1a–c. The truncation scheme comprises the residues 319–537, which contains the entire RBD with long loops at both the N- and C- termini. Besides that, according to the immune-escape capability and evolutionary convergence of the mutations carried by SARS-CoV-2 variants, the RBDs from the prototype, Beta, and Kappa strains, respectively, were selected for the construction of the hybrid immunogen. Compared with the prototype strain, the RBD of Beta variant harbors three residue mutations, i.e., K417N, E484K, and N501Y, and the RBD of Kappa contains L452R and E484Q mutations, respectively. Several of these mutations were also revealed to be evolutionarily convergent. Three truncated RBDs were then connected end-to-end to generate a heterologous tri-RBD, i.e., mutI-tri-RBD. The homo-tri-RBD constructed only based on the prototype strain was also produced as a comparison (Fig. 1a).

The possible tertiary structures of the designed mutI-tri-RBD and homo-tri-RBDs were built by the Modeller9.23 software^25^, using the native structure of S trimer as the template. To analyze the stereo-chemical rationality and stability of the modeled structures, the constructed trimeric structures were subjected to 200 ns all-atomic MD simulations with GROMACS software^26^. Simulation results showed that both modeled mutI-tri-RBD and homo-tri-RBD structures were stereo-chemically stable without serious steric clashes. After 200 ns MD simulations, three RBDs were arranged in a loosely-packed trimeric form both in mutI-tri-RBD and homo-tri-RBD (Fig. 1b and Supplementary Videos S1 and S2). It should be mentioned that we do not exclude the possibility that the connected three RBDs assembled into another trimeric form different from that in the native structure of S trimer, which needs further validation by the experimental determination of its three-dimensional structure in future research.

### Expression, purification, and characterization of the recombinant mutI-tri-RBD and homo-tri-RBD

The designed mutI-tri-RBD as well as homo-tri-RBD proteins of SARS-CoV-2 were transiently expressed in mammalian HEK293T cells. The culture supernatant of the transfected cells was harvested and purified by chromatography followed by ultrafiltration. Sodium dodecyl sulfate–polyacrylamide gel electrophoresis (SDS-PAGE) exhibited an obvious single band with the molecular mass of about 90 kDa both for the recombinant mutI-tri-RBD and homo-tri-RBD proteins (Fig. 1c), indicating the integrity of the trimeric RBDs for the designed two schemes. Protein purity was determined over 95% for both mutI-tri-RBD and homo-tri-RBD. Furthermore, for mutI-tri-RBD protein, the exact molecular weight was determined to be around 87,836 Da by matrix-assisted laser desorption ionization–time-of-flight mass spectrometer (MALDI-TOF MS) (Fig. 1d), which is larger than the theoretical value of about 74 kD calculated based on amino acid sequence. The difference between the experimental and calculated molecular weights is attributed to the dense glycosylation of the protein. After removing the glycans, the molecular weight of the recombinant mutI-tri-RBD was determined to be 73,929 Da by ultra-high performance liquid chromatography-mass spectrometer (UPLC-MS), which agrees well with the theoretical value (Fig. 1e).

Secondary structural composition analysis of the mutI-tri-RBD protein by circular dichroism (CD) illustrated that the recombinant protein was correctly folded with about 13.5% helix, 23.3% β strands, 11.4% turn, and 51.8% others (Fig. 1f and Supplementary Fig. S2a, b). For comparison, the secondary structures in an RBD monomer were further calculated by DSSP software^27,28^ based on the X-ray resolved structure (PDB accession code: 6m0j)^29^, showing a composition of 10.1%, 22.4%, 8.7%, and 58.8% for helix, β strands, turn, and others, respectively. The sequence of one RBD contains 8 cysteine residues, and in the native structure of RBD in S protein, these cysteines form four intradomain disulfide bonds. Disulfide-linkages mapping by LC-MS showed that all these four disulfide bonds in RBD native structure also correctly formed in the recombinant mutI-tri-RBD protein (Fig. 1g and Supplementary Fig. S3a, b). The secondary structure and disulfide bonds analyses suggested that each of the three RBDs in the recombinant mutI-tri-RBD protein was correctly folded into its native structure. Then the transition temperature (*T*_m_) was determined to be 47.2 °C by differential scanning calorimetry (DSC), demonstrating the stability of mutI-tri-RBD (Fig. 1h).

The bioactivity of the recombinant tri-RBD proteins was assessed using two anti-RBD monoclonal neutralizing antibodies (nAbs), i.e., MM43 and R117, by enzyme-linked immunosorbent assay (ELISA). MM43 can bind not only with the prototype RBD but also with the Beta and Kappa RBDs. High binding activities with MM43 were observed both for the recombinant mutI-tri-RBD and homo-tri-RBD proteins, indicating the formation of native conformation for individual RBD in the designed trimeric proteins (Fig. 1i). R117 binds only to the prototype and Kappa RBDs, but not to the Beta RBD. A lower binding activity to R117 was observed in mutI-tri-RBD than that of homo-tri-RBD, especially at low concentrations (Fig. 1i), which is within our expectation as Beta RBD is designed to be embedded in mutI-tri-RBD.

Then, the binding strength of the mutI-tri-RBD protein with the receptor hACE2 was also quantified by surface plasmon resonance (SPR) assay. Owing to the avidity effect of the trimeric form of mutI-tri-RBD, the binding strength was evaluated by the apparent dissociation constant *K*_D_, which was determined to be 3.20 × 10^−3^ nM, with the association rate constant *k*_a_ of 1.78 × 10^7^ M^−1^ S^−1^ and the dissociation rate constant *k*_d_ of 5.67 × 10^−5^ S^−1^ (Fig. 1j). SPR assay demonstrated that the designed mutI-tri-RBD protein binds specifically to hACE2 with high avidity, implying correct folding of the RBDs and high functionality of the recombinant mutI-tri-RBD protein. These results supported the mutI-tri-RBD to serve as an excellent immunogen.

### Both mutI-tri-RBD and homo-tri-RBD elicited a high level of immune responses against the SARS-CoV-2 prototype strain in mice

**Fig. 2:**
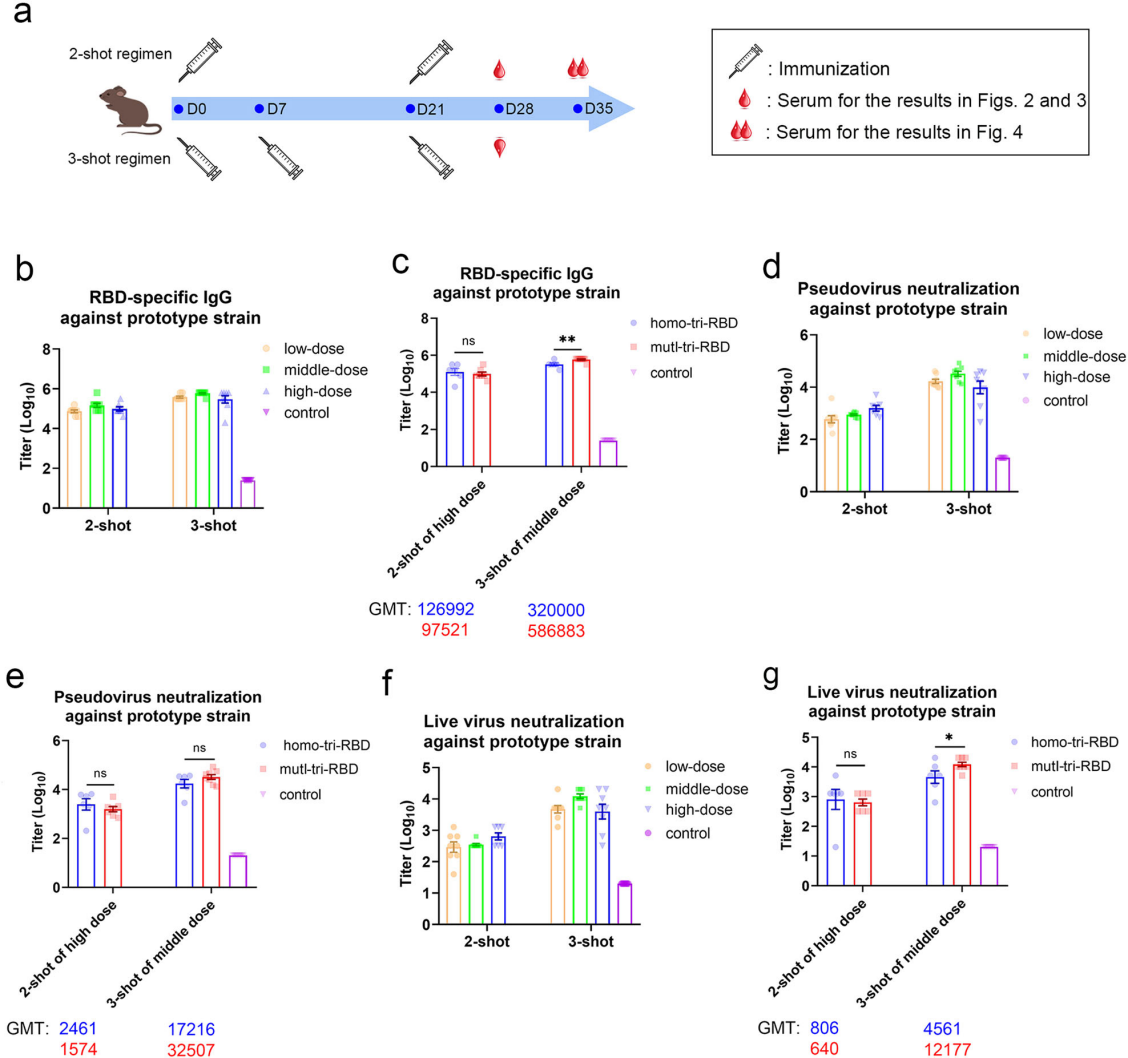
mutI-tri-RBD and homo-tri-RBD elicited similarly high levels of the immune response against the SARS-CoV-2 prototype strain in mice. The mice were immunized with two-shot or three-shot injections, and for each shot, three different doses were used, including low (0.125 µg/dose), middle (0.5 µg/dose), and high (2.0 µg/dose) doses, respectively. **a** The timeline of vaccine immunization and serum collection. The levels of specific IgG elicited by mutI-tri-RBD in the sera collected on day 7 post-immunization (D28 in **a**), were detected with ELISA by using monomeric RBD of prototype SARS-CoV-2 strain, and the titers of neutralizing antibodies against the prototype strain were assessed by using the pseudo- and live virus neutralization assays. **b**, **d**, **f** An obvious dose-dependent response of the RBD-specific IgG (**b**) as well as pseudo-virus (**d**) and live-virus (**f**) neutralizing antibodies induced by mutI-tri-RBD were observed. **c**, **e**, **g** The RBD-specific IgG level (**c**), and the neutralizing antibody titers against the pseudo (**e**) and live (**g**) viruses elicited by mutI-tri-RBD were similar to those elicited by homo-tri-RBD. Data are presented as means ± SEM. *P* values were calculated by using one-way ANOVA followed by Sidak’s multiple comparison test. *P < 0.05, **P < 0.01, ns, not significant.

The immunological effects of the trimeric RBDs were tested in BALB/c mice using aluminum as the adjuvant. Animals were immunized intraperitoneally with two or three shots as shown in Fig. 2a, and for each shot, three different doses, including low-dose (0.125 µg/dose), middle-dose (0.5 µg/dose) and high-dose (2.0 µg/dose), respectively, were applied. The level of RBD-specific IgG in the mice sera, collected on day 7 post-immunization (D28 in Fig. 2a), was measured by using ELISA. RBD monomer from the SARS-CoV-2 prototype strain was used to coat the wells of the ELISA plates. Three-injection immunization regimen elicited distinctly higher immune response compared with the two-injection treatment, where the anti-RBD IgG GMTs were improved by 5.2, 4.0, and 3.0 times for the low-, middle- and high-dose vaccinations, respectively (Fig. 2b and Supplementary Table S1). Both in the two-shot and three-shot regimens, middle-dose immunization elicited a higher level of anti-RBD IgG than low-dose, but no further increase was observed by high-dose vaccination (Fig. 2b and Supplementary Table S1). Furthermore, in order to compare the immunogenicity between mutI-tri-RBD and homo-tri-RBD, BALB/c mice were also immunized with the homo-tri-RBD by two-injection of high dose or three-injection of middle dose. The results showed that the RBD-specific IgG induced by the mutI-tri-RBD was comparable to that elicited by the homo-tri-RBD, indicating similar immunogenicity between two trimeric RBDs (Fig. 2c and Supplementary Table S2).

Then, the GMT of the neutralizing antibodies against SARS-CoV-2 prototype strain was assessed by using the pseudo- and live virus neutralization assays. The sera from the immunized mice potently neutralized both the pseudo- and live virus infections. Three shots of the mutI-tri-RBD induced significantly higher neutralizing antibody titers than two shots, as shown by 6.2–36.8 times higher against pseudo-virus and 6.2–34.9 times higher against live prototype virus (Fig. 2d, f and Supplementary Tables S3 and S5). Moreover, in animals immunized with the two-shot regimen, neutralizing GMT showed a dose-dependent increase in both pseudo- and live virus assays. In animals with the three-injection regimen, middle dose induced the highest neutralizing antibody titer, and the neutralizing GMT against live prototype virus reached as high as 12,177 (Fig. 2f, g and Supplementary Tables S5 and S6). More importantly, both in the two-shot and three-shot immunizations, the GMTs of neutralizing antibodies induced by mutI-tri-RBD were not less than those of homo-tri-RBD (Fig. 2e, g and Supplementary Tables S4 and S6). These results indicated that mutI-tri-RBD containing only one prototype RBD could elicit a similar response as homo-tri-RBD with three repeated prototype RBDs build-in.

The homo-tri-RBD has been approved by China National Medical Products Administration (NMPA) to enter a clinical trial. Here, the immune responses of IgG and neutralizing antibodies in mice suggested that the designed mutI-tri-RBD exhibited similar immunogenicity to the homo-tri-RBD against the SARS-CoV-2 prototype virus, although the former harbors only one-third prototype RBD in contrast to the latter. These results supported that the mutI-tri-RBD may also serve as an effective vaccine candidate against the SARS-CoV-2 prototype strain.

### mutI-tri-RBD induced significantly higher titer of neutralizing antibody responses against the Delta and Beta variants than homo-tri-RBD

We hypothesized that mutI-tri-RBD would be a vaccine candidate with broad neutralizing effects against SARS-CoV-2 variants. Delta, as a highly transmissible variant, is rapidly becoming the dominant strain in many countries and also exhibits reduced sensitivity to neutralizing antibodies present in convalescent patients and vaccinated individuals. The Beta variant has also been believed to possess the ability to evade pre-existing immunity elicited by natural infections and vaccinations. Therefore, we first tested whether the designed mutI-tri-RBD can elicit robust neutralizing antibody responses against these two variants. The titers of neutralizing antibodies against the Delta and Beta variants, induced by the mutI-tri-RBD were evaluated in the mice sera, collected on day 7 post-immunization (D28 in Fig. 2a), by using pseudo- and live virus neutralization assays, which were then compared with those induced by the homo-tri-RBD.

**Fig. 3:**
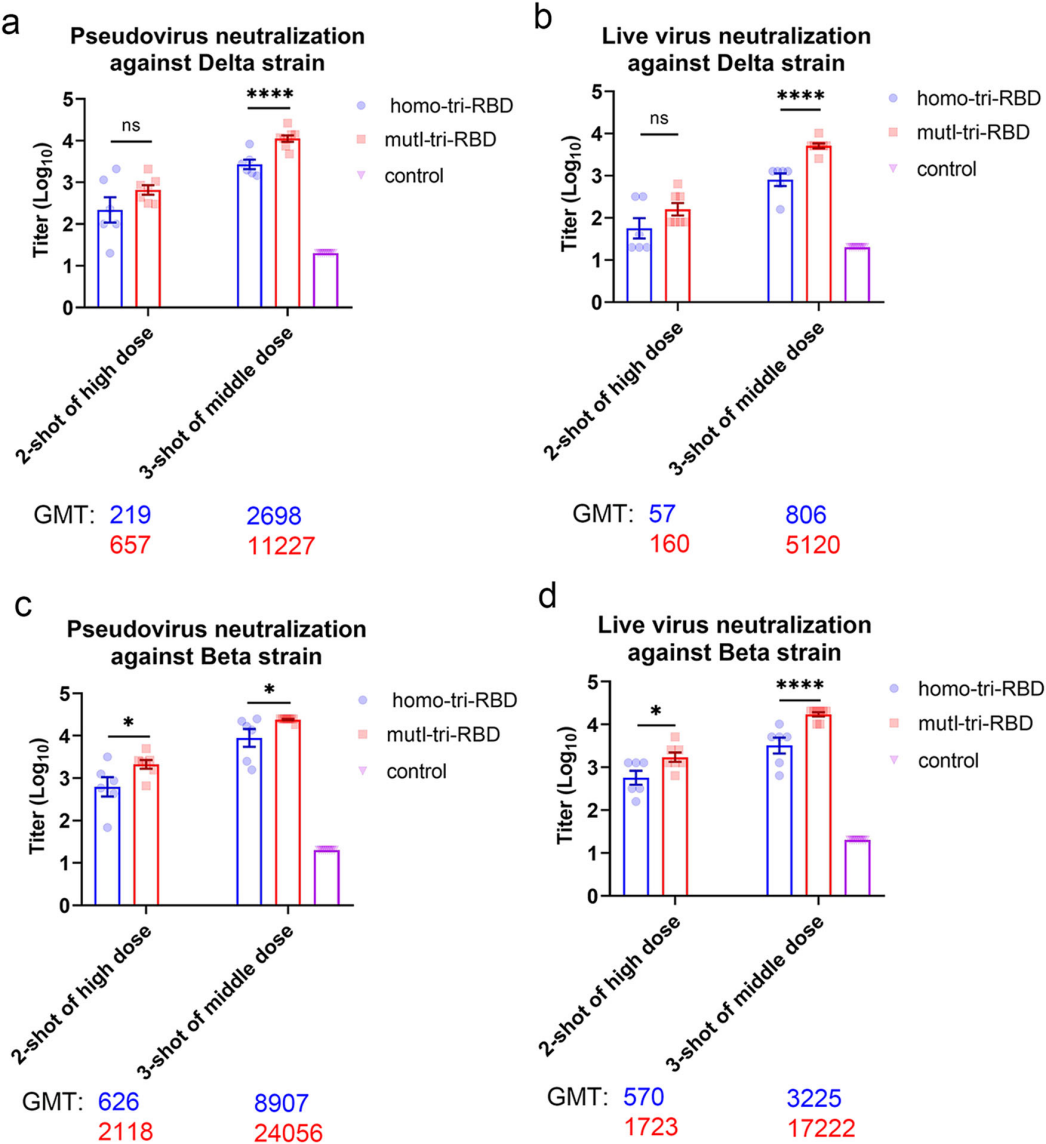
mutI-tri-RBD induced significantly higher titer of neutralizing antibody responses against the Delta and Beta variants compared with homo-tri-RBD. **a**, **b** The pseudo-virus (**a**) and live-virus (**b**) neutralizing antibody titers against the SARS-CoV-2 Delta variant induced by mutI-tri-RBD in the sera collected on day 7 post-immunization (D28 in Fig. 2a) were distinctly higher than those elicited by homo-tri-RBD. **c**, **d** The pseudo-virus (**c**) and live-virus (**d**) neutralizing antibody titers against SARS-CoV-2 Beta variant induced by mutI-tri-RBD were also obviously higher than those elicited by homo-tri-RBD. Data are presented as means ± SEM. *P* values were calculated by using one-way ANOVA followed by Sidak’s multiple comparison test. *P < 0.05, ****P < 0.001, ns, not significant.

Regarding the Delta variant, both mutI-tri-RBD and homo-tri-RBD induced elevated neutralizing antibody responses in the immunized mice; however, the neutralizing antibody levels stimulated by mutI-tri-RBD were significantly higher than those elicited by homo-tri-RBD both in pseudotype and live virus neutralization assays. The neutralizing antibody GMTs against the Delta pseudo-virus induced by mutI-tri-RBD were 3.0-fold and 4.2-fold higher than those induced by homo-tri-RBD in the two-shot and three-shot injections, respectively (Fig. 3a and Supplementary Table S7). Correspondingly, in the live virus neutralization tests, the anti-Delta neutralizing GMTs were improved by 2.8-fold and 6.4-fold, respectively, in the two-shot and three-shot vaccinations (Fig. 3b and Supplementary Table S8) for mutI-tri-RBD compared with those of homo-tri-RBD. Therefore, the mutI-tri-RBD exhibited superior immune efficacy than the homo-tri-RBD against the Delta variant.

Similar results were observed for neutralization assays on the Beta variant. Both two trimeric RBDs induced elevated neutralizing antibody responses; however, mutI-tri-RBD elicited significantly stronger neutralizing antibody responses against the Beta variant than homo-tri-RBD. The neutralizing GMTs against the pseudo-virus of Beta induced by mutI-tri-RBD were 3.4-fold and 2.7-fold higher compared with those induced by homo-tri-RBD in the two-shot and three-shot immunizations, respectively (Fig. 3c and Supplementary Table S9). The live virus neutralization assays further confirmed the pseudo-virus neutralization results, in which the neutralizing GMTs improved 3.0-fold and 5.3-fold, respectively, in the two-shot and three-shot vaccinations of the mutI-tri-RBD than those of the homo-tri-RBD (Fig. 3d and Supplementary Table S10). Particularly, a very high level of neutralizing antibody GMT of 17,222 against live Beta virus was observed in animals given with three-shots of mutI-tri-RBD (Fig. 3d and Supplementary Table S10). Thus, regarding the Beta variant, the immune efficiency of mutI-tri-RBD is distinctly better than that of homo-tri-RBD.

The above results demonstrated that the designed mutI-tri-RBD, which harbored RBD antigens from three circulating SARS-CoV-2 strains, not only maintained strong neutralizing antibody responses against the SARS-CoV-2 prototype strain but also induced significantly enhanced neutralizing antibody titers against the Delta and Beta variants than homo-tri-RBD.

### mutI-tri-RBD elicited broadly neutralizing activities against SARS-CoV-2 compared with homo-tri-RBD

Besides the SARS-CoV-2 prototype, Delta, and Beta strains, we also investigated whether the designed mutI-tri-RBD can also elicit broad neutralizing antibody responses against other emerging SARS-CoV-2 variants. The pseudo-viruses of nine other circulating strains, including D614G, 501Y.V2-1, 501Y.V2-3, Alpha (B.1.1.7), Gamma (P.1), Zeta (P.2), Epsilon (B.1.429B), Eta (B.1.525), and Iota (B.1.526), as well as 11 corresponding single or combinatorial mutants were tested^30–33^, in pseudo-virus neutralization assays. Due to a large amount of serum needed, serum samples were re-collected on day 14 post-vaccination (D35 in Fig. 2a) from the mice immunized with two shots of high dose of mutI-tri-RBD or homo-tri-RBD. The prototype, Delta, and Beta strains were also re-tested by using the 14-day post-immunization sera. In order to assess whether the mutI-tri-RBD has a superior effect in inducing broad neutralization against these SARS-CoV-2 strains than the homo-tri-RBD, the GMT values elicited by the mutI-tri-RBD were compared with those induced by the homo-tri-RBD.

**Fig. 4:**
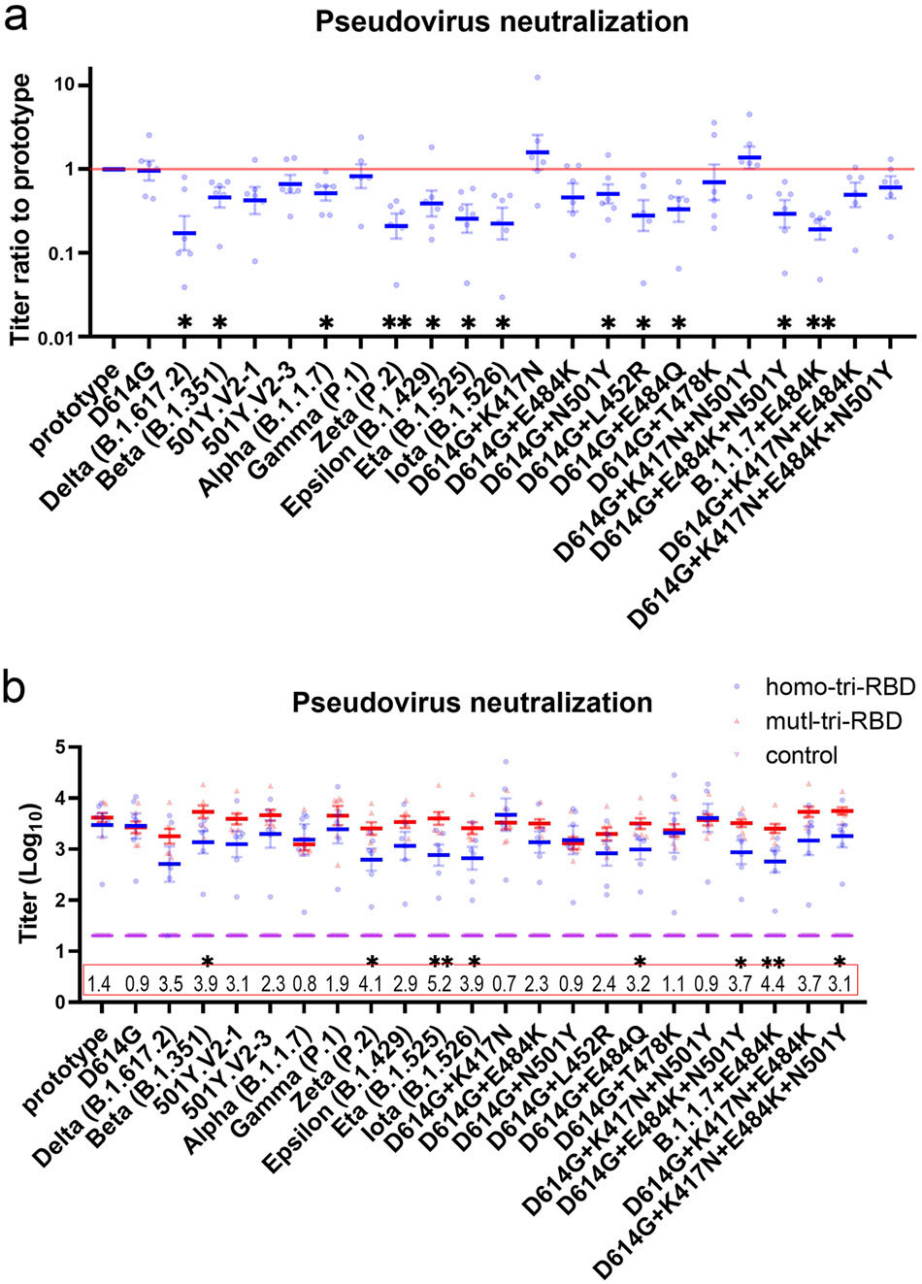
The neutralizing antibody responses against 23 various SARS-CoV-2 pseudo-virus strains elicited by mutI-tri-RBD were significantly stronger than or comparable to those induced by homo-tri-RBD. **a** Sensitivities to the neutralization of homo-tri-RBD immunized sera for the various pseudo-typed variants compared with that of the prototype strain. The sera collected on day 14 post-immunization (D35 in Fig. 2a) were used in the pseudo-virus neutralization assays. In this figure, the titers of the prototype strain are taken as a reference, and the titer ratios between the variants and the prototype strain are displayed. Each serum sample is presented as a dot in the plot, and each serum sample was tested against all these variants. Data are presented as means ± SEM. *P* values were calculated with Student’s *t* test. *P < 0.05, **P < 0.01. **b** Neutralizing antibody GMTs against the various pseudo-typed strains induced by mutI-tri-RBD (red color) compared with those elicited by homo-tri-RBD (blue color). Numbers in the red box indicate the GMT ratios of mutI-tri-RBD to homo-tri-RBD. Data are presented as means ± SEM. *P* values were calculated by using one-way ANOVA followed by Sidak’s multiple comparison test. *P < 0.05, **P < 0.01.

We first examined whether the 22 tested SARS-CoV-2 variants exhibited resistance to the neutralization elicited by homo-tri-RBD. Neutralization experiments showed that although homo-tri-RBD can induce neutralizing antibodies against all these tested variants in comparison with the control group, some variants also displayed obviously reduced sensitivities to the neutralization of the immunized sera compared with the prototype strain. Among the eleven circulating variants, Delta, Beta, Alpha, Zeta, Epsilon, Eta, and Iota exhibited significantly decreased neutralization susceptibilities by varying extents (Fig. 4a). Notably, all these variants harbored E484K, L452R, or N501Y mutation, suggesting the importance of these residues for immune resistance. This is further demonstrated by the corresponding single and combinatorial mutants of E484K, L452R, or N501Y, as most of them also displayed distinctly reduced neutralization sensitivities (Fig. 4a). In addition, the single mutation of E484Q also exhibited obviously reduced neutralization sensitivities to the antibodies elicited by homo-tri-RBD. However, the neutralization reactions against the K417N and T478K single-mutants were not reduced, implying that these two residues do not lead to immune resistance (Fig. 4a). This is consistent with the experimental results from another work, where K417N mutation leads to enhanced neutralization activity^31^.

Then, we tested whether the designed mutI-tri-RBD can induce more robust neutralization activities against these 22 SARS-CoV-2 variants than homo-tri-RBD, especially the homo-tri-RBD-resistant variants discussed above. The results showed that in almost all tested strains, neutralization GMTs induced by mutI-tri-RBD were comparable to or significantly higher than those induced by homo-tri-RBD. For E484K- or L452R-carrying variants, including Delta, Beta, 501Y.V2-1, 501Y.V2-3, Gamma, Zeta, Epsilon, Eta, and Iota, the neutralization activities induced by mutI-tri-RBD were improved by 1.9 to 5.2-fold in contrast to those induced by homo-tri-RBD (Fig. 4b and Supplementary Information, Table S11). For the E484K, L452R, as well as E484Q single mutations, the neutralization sensitivities elicited by the mutI-tri-RBD were raised 2.3-, 2.4-, and 3.2-fold, respectively, (Fig. 4b and Supplementary Table S11). Most importantly, as discussed above, the E484K, L452R, and E484Q mutations lead to obvious resistance to the sera immunized with homo-tri-RBD, which may partly reduce the efficacy of the homo-tri-RBD vaccine candidate. However, encouragingly, the mutI-tri-RBD enables a substantial increase of the neutralizing titers against these homo-tri-RBD-resistant variants, indicating potently broad neutralizing capabilities induced by mutI-tri-RBD (Fig. 4b and Supplementary Table S11). E484K has been believed to be the most important “immune escape” mutation, and our pseudo-virus neutralization assays displayed that the mutI-tri-RBD also induced robustly higher neutralizing antibody responses against the E484K-carrying combinatorial mutants in comparison with the homo-tri-RBD.

Many studies have demonstrated that the neutralizing antibody level is highly correlated with the protective efficacy, in which the higher the neutralizing antibody titer is, the lower the chance of breakthrough infection^34–37^. Therefore, the neutralizing antibody titer against different SARS-CoV-2 variants is a crucial indicator to evaluate the broadly protective ability of the vaccine. Our experimental results show that compared with homo-tri-RBD, mutI-tri-RBD elicited much higher neutralizing antibody titers against most of the tested SARS-CoV-2 variants, which indicates that it may potentially serve as a more promising broad-spectrum vaccine candidate against SARS-CoV-2 for further clinical developments.

### mutI-tri-RBD efficiently protected hACE2-transgenic mice from challenges with the SARS-CoV-2 prototype, Delta, and Beta live viruses

**Fig. 5:**
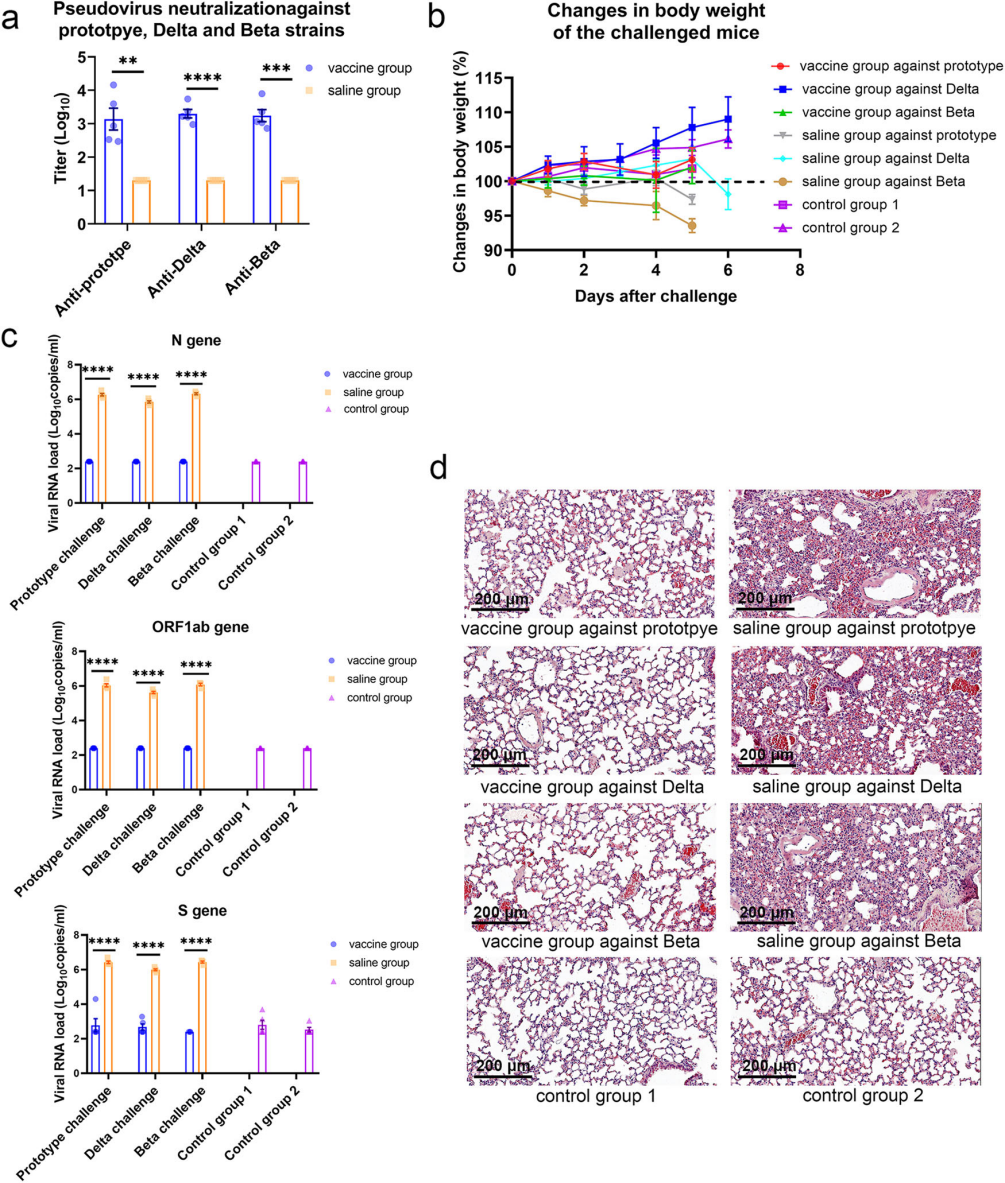
Protective efficacy of mutI-tri-RBD in hACE2-transgenic mice against challenge with the SARS-CoV-2 prototpye, Delta, and Beta strains, respectively. **a** The mice were immunized with two doses of mutI-tri-RBD with 2 µg/dose on Day 0 and Day 21, or injected with two doses of physiological saline as a control. On day 7 after the complete immunization, the sera from the caudal vein of the immunized mice were collected. The titers of pseudo-virus neutralizing antibodies in the sera of the immunized mice against the prototype, Delta and Beta SARS-CoV-2 strains, respectively, were measured for the vaccine groups and compared with those in the corresponding saline-treated groups. Data are presented as means ± SEM. *P* values were calculated with Student’s *t-*test. **P < 0.01, ***P < 0.001, ****P < 0.0001. **b** On day 14 or day 19 after the complete immunization, the mice were challenged with the prototype, Delta and Beta live SARS-CoV-2 viruses (1.5 × 10^5^ TCID_50_), respectively, and correspondingly control groups were challenged with saline (15 µL) as blank controls. The changes in the body weight of the mice were recorded during virus challenge experiments. Data are presented as means ± SEM. **c** The lung tissues were collected at 5 or 6 days after virus challenge. The viral load was measured by the copies of N, ORF1ab, and S genes. The viral load less than the detectable limit (<500 copies/μL) was set to half the value of the limit, i.e., 250 copies/mL. Data are presented as mean ± SEM. *P* values were calculated by using one-way ANOVA followed by Sidak’s multiple comparison test. ****P < 0.0001. **d** Histopathological examinations of the lung tissues of the mice. Scale bars, 200 μm (20×).

In order to validate the broad-spectrum protective ability of the designed mutI-tri-RBD in vivo, its protective efficacy was assessed in hACE2-transgenic mice against live virus challenges with three major SARS-CoV-2 strains, including the prototype, Delta and Beta. The mice in vaccine groups were immunized with two doses of mutI-tri-RBD with 2 µg/dose on Day 0 and Day 21, and correspondingly the mice in the saline groups and control groups were treated with two doses of physiological saline. On Day 7 after the whole vaccination, high titers of neutralizing antibodies were detected for all mice in vaccine groups, whereas in the corresponding saline groups, no neutralizing activity was observed (Fig. 5a and Supplementary Table S12). On day 14 or day 19 after the complete immunization, the mice in vaccine groups and saline groups were challenged with the live virus of the prototype, Delta and Beta SARS-CoV-2 strains, respectively. The mice in control groups were challenged with saline as blank controls. During the live virus challenge experiment, a mouse in the saline group challenged with Beta strain died on the fifth day. All the other mice survived, but muscle weakness and vitality decrease began to appear from the third day for the mice in all the saline groups challenged with the prototype, Delta or Beta strains. Especially, the mice in the saline group challenged with Beta virus experienced an obviously progressive drop in body weight. For the mice in all the vaccine groups, no infection-related symptoms and body weight loss were observed when compared with the control group (Fig. 5b and Supplementary Table S13).

Viral load in mice lung tissues was detected qPCR of N, ORF1ab, and S genes. All animals in saline groups showed high viral RNA loads after the live virus challenge. On the contrary, negative (<500 copies/mL) or very low viral loads were monitored in lung tissues for all the mice in the vaccine groups challenged with the prototype, Delta or Beta viruses (Fig. 5c and Supplementary Table S14). Histopathological examination of the lung tissues showed that severe interstitial pneumonia, a typical feature of COVID-19, occurred in the mice of the saline groups. Substantial histopathological changes were observed, including alveolar septa thickening, heavy inflammatory cell infiltration, and focal hemorrhage. Large amounts of exudates appeared in lung cavities, as well as the formation of hyaline membrane and bullae were also observed. In addition, tracheal bronchus and blood vessels also exhibited a severe infiltration and exudates combined with desquamation of epithelial cells. In contrast, no obvious histopathological change was observed in the lung tissues of the mice in all the vaccine groups challenged with the prototype, Delta or Beta SARS-CoV-2 viruses. The mice receiving vaccination showed normal lung tissue with only focal mild inflammatory infiltrations, which were similar to the mice in the control groups but significantly different from the results in the above saline groups (Fig. 5d). The animal challenge experiments indicated that mutI-tri-RBD vaccine candidate provided efficient broad protection not only against the prototype SARS-CoV-2 virus but also against the Delta and Beta variants.

## Discussion

RBD is directly involved in the recognition of SARS-CoV-2 to host cells, which is immunodominant in eliciting neutralizing antibody responses. As of October 2021, at least 23 RBD-based vaccine candidates are under clinical trials, among which a dimeric RBD-based protein subunit vaccine, ZF2001, has been authorized for emergency use^38^. Preclinical and clinical data revealed that RBD-based immunogen was not inferior to the full-length S protein in immunicity^39^. Clinical trial results showed that the RBD-dimer ZF2001 vaccine induced twofold higher neutralizing antibody GMTs than that of human convalescent sera (HCS)^40^, and the neutralizing GMTs yielded by the RBD-based nanoparticle vaccines were five times higher than HCS^41^. For the homo-tri-RBD vaccine candidate developed by our group, the preliminary results in the phase I/II clinical trial showed that neutralizing antibody GMTs were about 3–5-fold higher than those of HCS (data not yet published). Although RBD-based vaccines exhibited relatively robust and broad immunogenicity, the neutralizing efficacy against some of the SARS-CoV-2 variants was declined to a certain extent^42,43^.

The new waves of COVID-19 caused by SARS-CoV-2 variants and the rising number of breakthrough infections highlight the need of developing broadly protective vaccines to combat the present and future SARS-CoV-2 variants. Naturally trimeric arrangement of RBDs in S protein and its unique structural characteristics allow construction of heterogenous mutI-tri-RBD as a broadly protective vaccine candidate, in which the RBD antigens derived from different SARS-CoV-2 circulating strains were integrated into a single immunogen. The designed mutI-tri-RBD induced broadly neutralizing antibody responses against various circulating SARS-CoV-2 strains. Compared with the homo-tri-RBD, the hybrid mutI-tri-RBD displayed broader and superior neutralization efficacy against SARS-CoV-2. The broadly protective ability of mutI-tri-RBD was validated by using live virus challenge experiments, in which it not only potently protected the animals from the infections of the SARS-CoV-2 prototype strain but also provided efficient protection against the highly infectious Delta and the so-called “immune escape” Beta variants.

The anti-Beta vaccine candidate has recently been tested for a bivalent vaccination strategy in combination with the anti-prototype one or for heterologous prime boosting immunization strategy^44–46^. Here, we provided another promising strategy for the construction of a vaccine candidate with broad neutralizing capability by incorporating different variant-specific antigens and key mutations into one hybrid immunogen. Compared with the conventional multivalent strategy, our method has the advantages of broader protection capability, lower time costs, and higher production benefits.

Evolutionary analysis indicated that most of the immune-escape mutations integrated into mutI-tri-RBD are also evolutionarily convergent and considered to be of adaptive advantage, suggesting the possible recurrence of them individually or combinedly in the future^14^. Our results demonstrated that the designed mutI-tri-RBD induced a broader and robust neutralizing response against these evolutionarily-convergent and immune-resistant mutations, suggesting that our hybrid immunogen construct may have the potential to provide neutralizing capability against future-emerging SARS-CoV-2 variants.

Hybrid immunogen design strategy was also adopted by other groups to develop broadly protective vaccines against SARS-CoV-2 variants and other coronaviruses. Cohen et al.^47^ and Walls et al.^48^ constructed mosaic nanoparticle immunogens, in which multivalent RBDs from various coronavirus were co-displayed on the same particle surface. Owing to the high-dense and heterotypic arrays of the antigens, this mosaic nanoparticle-type vaccine candidate elicited much stronger and broader antibody responses compared with the monomeric immunogen. It was also immunogenically superior to the homotypic RBD nanoparticles. However, it is challenging for precise control of the relative number and distribution of different RBDs coated on the nanoparticle surface. In addition, the nanoparticle scaffold might also have unknown safety risks. Martinez et al.^15^ designed a chimeric S protein immunogen, in which the NTD, RBD, and S2 domains derived from different sarbecoviruses were integrated into a single molecule. Although the chimeric immunogen induced broadly protective antibodies against diverse sarbecoviruses, the construction strategy is not applicable for the assembly of different RBDs into a single immunogen. Compared with these approaches, the mutI-tri-RBD designed by our group enables the integration of multiple immunodominant RBDs into a single immunogen to improve and broaden immunogenicity. In our method, no exogenous sequence was introduced, which ensured the safety profile of this vaccine candidate.

Structure-guided design of new immunogen that integrates key immune-relevant mutations is an effective strategy for the development of vaccines with broadly protective capabilities, which can be used for other highly variable viruses besides SARS-CoV-2. This mutation-integration strategy can be realized not only in protein subunit vaccine developments but also in mRNA and DNA vaccine platforms.

After we have submitted the manuscript, during the review process, SARS-CoV-2 Omicron is growing rapidly to be the most prevalent variant. A randomized clinical trial for mutI-tri-RBD has been conducted in the United Arab Emirates, and the neutralizing activity against Omicron variant induced by the vaccine will be evaluated in the trial. Currently, the trial is still ongoing, and we will report the data in the near future.

## Materials and methods

### Structure modeling and MD simulations

To construct mutI-tri-RBD, the RBD region (residues 319–537) was truncated from the S proteins of the prototype, Beta (B.1.351) and Kappa (B.1.617.1) SARS-CoV-2 strains, respectively. Then these three RBDs were connected end-to-end by using their own long loops at the N- and C-terminus without introducing exogenous linker. As a comparative control, the homo-tri-RBD was also constructed in which the three RBDs were all derived from the prototype strain. The possible three-dimensional structures of the designed mutI-tri-RBD and homo-tri-RBD proteins were modeled with the Modeller9.23 software^25^ by using the native structure of S trimer (PDB accession code 6zgi for homo-tri-RBD, as well as 6zgi and 7lyl for mutI-tri-RBD) as the template. A total of 10 structures were generated both for mutI-tri-RBD and homo-tri-RBD, and the structure with the lowest value of the DOPE assessment score^49^ was picked as the best model.

Then, a 200 ns atomic MD simulation was performed both for the mutI-tri-RBD and homo-tri-RBD. All the MD simulations were carried out by using Gromacs 2019 with the Charmm27 force field^26^. The trimeric structure generated by Modeller^25^ was solvated using SPC water molecules in a cubic box, with the protein atoms being at least 1.6 nm away from the box edges. A total of 24 and 21 CL^-^ ions were added into the water box to neutralize the net charges of the simulation systems for the mutI-tri-RBD and homo-tri-RBD, respectively. The prepared system was then subjected to energy minimization with the steepest descent algorithm to ensure that the maximum force in the system was below 1000 kJ/mol/nm. After energy minimization, a 100 ps NVT simulation with position restraints on protein atoms was performed at 300 K, which was followed by a 100 ps NPT simulation with position restraints. Finally, the production simulation without any position restraint was run for 200 ns and the snapshots were collected every 10 ps to obtain the simulation trajectory. In the simulation, a time step of 2 fs was used and all H-bonds were constrained with LINCS algorithm. Both in the mutI-tri-RBD and homo-tri-RBD, each RBD unit contains four disulfide bonds, and these disulfide bonds were included in the construction of the topology files for the simulation systems. A cutoff value of 1.0 nm was used for the calculation of both short-range electrostatic and van der Waals interactions. Long-range electrostatic interactions were computed by using the Particle-Mesh Ewald (PME) algorithm. During the simulation, the temperature and the pressure of the system were maintained at 300 K and 1 bar with the velocity rescaling and the isotropic Parrinello-Rahman coupling methods, respectively. Based on the MD simulation trajectories, the changes in the root mean square deviation of the C_α_ atoms of the system as a function of time were calculated by using the built-in tool of “gmx rms” in Gromacs to evaluate the stability of the trimeric RBD proteins, and the motion movies were generated by using Chimera software^50^ to display the intrinsic conformational movements of the trimeric RBD proteins.

### Protein expression and purification

The gene sequences of the designed mutI-tri-RBD and homo-tri-RBD proteins were codon optimized for the transient expression in the mammalian cells. In the designed trimeric schemes, mutI-tri-RBD is composed of three RBD regions derived from the S proteins of the prototype, Beta and Kappa SARS-CoV-2 strains, respectively, connected end-to-end with each other. Homo-tri-RBD consists of three copies of the RBD region from the prototype strain. For gene cloning of these designed proteins, signal peptide and Kozak sequences were added to the N-terminal of the expressed protein sequences, which were then inserted into the PTT5 plasmid via the *Hin*dIII and *Not*I restriction sites to construct the recombinant plasmids for the expression of the trimeric RBDs in HEK293T cells. The sequences of the constructed plasmids for the mutI-tri-RBD and homo-tri-RBD proteins were verified by gene sequencing. Then, the generated plasmids were transfected into the HEK293T cells for transient expression. After 3–5 days of culture, the culture supernatants from the transfected cells were harvested and purified by chromatography. During the chromatographic purifications, the isolated proteins from the eluted peaks were analyzed with SDS-PAGE. Then, the recovered protein sample was finally purified by ultrafiltration with the membranes of 30 kDa molecular weight cutoff. The purity of the produced proteins was determined by the size exclusion chromatography–high-performance liquid chromatography (SEC-HPLC) using TSKgel G2500PW column.

### Protein molecular weight measurement

MALDI-TOF-MS analysis was carried out with AB Sciex 4800 Plus MALDI TOF analyzer to measure the molecular weight of the recombinant mutI-tri-RBD^51,52^. The protein sample was exchanged into the water to the concentration of 5 mg/mL, and 0.5 µL of the sample was spotted onto the MALDI target plate, followed by air drying at room temperature. The MALDI plate was then covered with 0.5 µL of 0.5 mg/mL sinapinic acid (SA) dissolved in 0.1% TFA and 50% CAN, and also dried by air at room temperature. The MALDI TOF/TOF analyzer was equipped with a Nd:YAG 355 nm laser. Parameters were set as follows: linear mode; repetition rate laser: 200 Hz; mass range: 20,000–150,000 Da; 400 shots accumulated per profile.

### Measurement of protein molecular weight after deglycosylation

Firstly, the N- and O-linked glycans were removed from the protein samples. 100 µg of the sample was mixed with 5 µL 10× Deglycosylation Mix Buffer 1 (B6044S) and 5 µL Protein Deglycosylation Mix II (P6044S), which was then incubated at the room temperature for 30 min and further at 37 °C for 16 h. After that, the fully deglycosylated protein was separated by UPLC with Thermo Fisher Scientific UltiMate 3000 system. UHPLC was performed using a Nano-Mico UniPS 3–300 Column (3 μm, 2.1 mm × 50 mm) with a mobile phase of 0.1% formic acid (FA)-water and 0.1% FA-acetonitrile. The temperatures of the sample chamber and the column were respectively maintained at 4 and 60 °C, and the flow rate was set to 0.4 mL/min. Then, the eluting sample was analyzed using Thermo Fisher Scientific Q Exactive Plus mass spectrometer (MS), in which the MS spectra were acquired in positive ion mode for 8 min with a scan range of 1000–4000 m/z and a resolution of 17,500. The acquired raw data files were analyzed with BioPharma Finder software to calculate the molecular weight.

### Disulfide bond identification

To identify the disulfide bond locations in the recombinant mutI-tri-RBD protein by using the UPLC-MS peptide mapping method, both reduced and non-reduced protein samples were prepared. In sample preparation, the NEM solution was firstly added into the sample to block free sulfhydryl groups in the protein at room temperature for 2 h. Then, the protein was transferred into a digestion buffer and pre-cleaved with Lys-C at 37 °C for 4 h followed by additional digestion with Glu-C at 37 °C overnight. Half of the digested sample was taken as the non-reduced sample, and the other half was further subjected to reduction and alkylation to prepare the reduced sample. In reduction and alkylation reactions, the digested peptides were reduced with DTT at 37 °C for 30 min and alkylated with IAA in the dark at room temperature for an additional 30 min. Then the resulted peptides were separated both for the reduced and non-reduced samples by UPLC using a C18 column (130Å, 1.7 μm, 2.1 mm × 150 mm) with the column temperature of 60 °C, and the reversed-phase chromatography of the sample was performed for 95 min with a flow rate of 0.3 mL/min and a UV detection wavelength of 214 nm. Subsequently, the separated peptides were detected and analyzed by Q Exactive Plus MS in positive ion mode, with *m*/*z* range of 300–2000 and a resolution (Full MS/MS2) of 35,000/17,500. The MS raw data files were processed using BioPharma Finder software (version 3.2). The presence of disulfide bonds containing peptides was determined through comparative analysis of the reduced and non-reduced peptide samples and were shown as indicated in the chromatogram as shaded peaks. The free sulfhydryl of a certain cysteine was quantified through the ratio of MS area of the NEM modification containing peptides against MS area of all peptides in the reduced sample.

### Determination of secondary structural composition

Protein secondary structural composition was determined by using CD spectroscopy, which was performed on Applied Photophysics following the operation procedure provided by the manufacturer. The protein sample was dialyzed into the buffer composed of 5 mM PB, and then placed to the spectrophotometer cell. Both far-ultraviolet (UV) and near-UV spectra were acquired in the wavelength range of 190–250 and 250–304 nm, respectively, with a spectral resolution of 0.5 nm and bandwidth of 1.0 nm. All CD spectra were obtained with 0.5 S per point at 25 °C. Both the spectra of the sample and the buffers under the same experimental conditions were recorded, and the actual protein spectra were obtained by subtracting the spectra of the buffers. The scan measurement was repeated 6 times and the average result was calculated to ensure the reliability of the measurement. The acquired CD data were analyzed with the BeStSel Server (http://bestsel.elte.hu/index.php)^53^ to estimate the content of different secondary structures in the protein.

### Protein stability characterization

The thermal stability of the recombinant mutI-tri-RBD was characterized by using a DSC assay. The protein sample was prepared at the concentration of 2.0 mg/mL in origin formulation buffer and 0.3 mL of the sample was used to perform the DSC experiment with the TA Instrument Nano-DSC. The thermograms were obtained over the temperature range from 5 to 95 °C using a scan rate of 1 °C/min. The experimental data were processed by Launch NanoAnalyze software supplied with the DSC instrument.

### SPR assay

The binding avidity of mutI-tri-RBD with receptor hACE2 was measured by using surface plasmon resonance (SPR), which was carried out by BIAcore 8 K (GE Healthcare) with NTA chips. The His-tagged receptor protein hACE2 (Sino Biological Inc., China. Cat: 10108-H08H) was dissolved in HBS-T buffer (HBS buffer and 0.05% Tween20) and immobilized onto the NTA chip. The protein sample was diluted with HBS-T buffer at the concentrations of 0.0173, 0.0346, 0.0692, 0.1385, and 0.277 µg/mL, which were then flowed over the chip surface at a rate of 30 µL/min for 120 s. The bound protein was then dissociated for an additional 120 s. During the association and dissociation processes, the real-time response SPR signal was recorded. After that, the sensor chip was regenerated using 350 mM EDTA regeneration solution for 120 s with a flow rate of 30 µL/min. The recorded data were processed using BIAcoreTM Insight Evaluation software and the binding kinetics were analyzed with a 1:1 binding model to obtain the association rate constant *k*_a_, the dissociation rate constant *k*_d_, and the apparent dissociation constant *K*_D_.

### Evaluation of nAb-binding activities

The binding activities of the recombinant mutI-tri-RBD and homo-tri-RBD proteins with two monoclonal nAbs, including MM43 and R117 (Sino Biological Inc., China. Cat: 40591-MM43 and 40592-R117), were evaluated by using ELISA. As controls, the binding activities with the monoclonal nAbs for the monomeric his-tagged RBDs from the prototype, Beta (B.1.351) and Kappa (B.1.617.1) SARS-CoV-2 strains were also measured. To detect the binding activity with the anti-RBD monoclonal nAbs, the protein samples were prepared at the starting concentration of 1.0 µg/mL, which were then subjected to 2-fold serial dilutions and coated on the 96-well microplate with 100 µL per well at 2–8 °C overnight. Subsequently, the plate was washed 3 times with phosphate-buffered saline (PBS) containing 0.05% Tween 20 (PBST) and then blocked with 100 µL blocking buffer per well, followed by incubation at 37 °C for 2 h. After washing the plate 3 times with PBST, the nAb sample was diluted to 1.0 µg/mL and added into the wells with 100 µL per well, which was then incubated at 37 °C for 1 h. After that, the plate was washed 3 times with PBST and the horseradish peroxidase (HRP)-conjugated goat anti-mouse or goat anti-rabbit IgGs at 1:10,000 dilution was added into the wells of the plate. The plate was again incubated at 37 °C for 1 h and washed 3 times with PBST. Subsequently, 50 µL tetramethylbenzidine (TMB) and 50 µL hydrogen peroxide solutions were added to start the color reaction. After color development for 5 min, the reaction was stopped using 0.2 M sulfuric acidic solution with 50 µL per well, and absorbance at 450 nm was read by plate reader. The background absorbance at 630 nm was also measured, and then the difference between absorbance at 450 nm and 630 nm (OD_450/630nm_) was obtained to detect the specific binding of the mutI-tri-RBD and homo-tri-RBD proteins with the nAbs.

### Ethics statement

All the animal experimental procedures were carried out according to Chinese animal use guidelines and were approved by the Institutional Animal Care and Use Committee (IACUC) of the National Vaccine and Serum Institute (NVSI) of China. All challenge experiments with live SARS-CoV-2 were approved by the IACUC of NVSI of China, and the IACUC of National Institute for Viral Disease Control and Prevention, Chinese Center for Disease Control and Prevention, Beijing, China.

### Mouse immunization protocols

To evaluate the impacts of different immunization regimens as well as vaccination doses on the immune responses, a total of 6 groups of female specific pathogen-free (SPF) mice (purchased from Beijing Vital River Laboratory Animal Technology Co., Ltd., China.) with 8 mice in each group, aged 6–8 weeks, were intraperitoneally vaccinated with the mutI-tri-RBD vaccine candidate mixed with aluminum hydroxide adjuvant. In these experimental groups, the mice were immunized with two shots on day 0 and day 21, or with three shots on Day 0, Day 7, and Day 21, respectively. For each shot, three different doses, including low dose (0.125 µg/dose), middle dose (0.5 µg/dose), and high dose (2.0 µg/dose), respectively, were used. Another 8 mice were injected with only adjuvant on Day 0, Day 7, and Day 21, serving as the control group. In order to compare the immunogenicity of the mutI-tri-RBD with the homo-tri-RBD, another two groups of BALB/c mice (6 mice in each group) were also immunized with the homo-tri-RBD plus aluminum hydroxide adjuvant by two shots of high dose (2.0 µg/dose) or three shots of middle dose (0.5 µg/dose). For all these groups, the sera of the immunized mice were collected at 7 days and 14 days after the last immunization.

### Mouse serum IgG measurement

The titer of the RBD-specific IgG in the serum of the immunized mice was measured with ELISA by using monomeric His-tagged RBD of the SARS-CoV-2 prototype strain. His-tagged RBD was diluted with coating buffer to the concentration of 1 µg/mL, which was coated on the 96-well plate with 100 µL per well at 2–8 °C overnight. After washing 3 times with PBST, the plate was blocked using blocking buffer with 100 µL per well and incubated at 37 °C for 2 h. Then, the plate was washed 3 times with PBST, and the serum samples were diluted by 2-fold serial dilutions and added into the well with 100 µL per well. After incubating the plate at 37 °C for 1 h and washing 3 times with PBST, the HRP-conjugated goat anti-mouse IgG at 1:10,000 dilution was added into the wells of the plate. The plate was again incubated at 37 °C for 1 h and washed 3 times with PBST. Subsequently, a color reaction was performed for 10 min by adding the color development solution, which was then stopped using a sulfuric acidic solution. Both absorbances at 450 and 630 nm were measured and the difference between them OD_450/630nm_ was obtained. To evaluate the titer of RBD-specific antibodies, the OD_450/630nm_ value of the well without adding serum sample was taken as the blank control, and 2.1 times of the control value was used as the cutoff for positive results. The titer of RBD-specific IgG was determined as the reciprocal of the maximum dilution of the serum, where the OD_450/630nm_ value is equal to or greater than the cutoff value.

### Pseudo-virus neutralization assay

A total of 23 SARS-CoV-2 pseudo-viruses were used to evaluate the neutralizing antibody titers in the sera from immunized mice. The SARS-CoV-2 pseudotyped viruses used in the studies were constructed by using a vesicular stomatitis virus (VSV)-base system^30–33^. To evaluate the neutralization activities of the sera against these pseudo-viruses, the serum sample was serially diluted fivefold at the starting dilution of 1:40 with the cell culture medium (DMEM containing 10% Fetal Bovine Serum, 25 mM HEPES and 1% penicillin–streptomycin). The serially diluted serum samples were added to the well of the plate with 50 µL per well. The SARS-CoV-2 pseudo-virus was diluted 9 times with the cell culture medium to the titer of 1.3 × 10^4^ TCID_50_ per mL and added into the well using 50 µL per well to mix with the serum, which was then incubated at 37 °C for 1 h. 50 µL per well medium was also mixed with the pseudo-virus as control. 100 µL per well medium served as blank. The HuH-7 cells were digested and suspended in the culture medium with a viable cell density of 2 × 10^5^ per mL, which were then added into the well with 100 µL per well. After culture with 5% CO_2_ at 37 °C for 20–24 h, the culture medium was removed, the cells were lysed, and the luciferase activity was evaluated as the relative light unit value. The neutralizing antibody titer was determined as the reciprocal of the dilution of the serum for 50% neutralization of viral infection, which was calculated using the Reed–Muench method.

### Live SARS-CoV-2 virus neutralization assay

To evaluate the neutralization activity against the live SARS-CoV-2 virus, the live virus neutralization assay was performed in a BSL-3 laboratory of Guangdong Provincial Center for Disease Control and Prevention. Both the prototype (2020XN4276 strain), Delta (2020K-XG0186 strain), and Beta (20SF18530 strain) viruses were used in the live virus neutralization assays. The mouse serum was serially diluted and mixed with an equal volume of 100 TCID_50_ live SARS-CoV-2 virus. After incubation at 37 °C for 2 h, the serum-and-virus mixed solution was added into the well of the plate containing Vero-E6 cells with a density of 2 × 10^5^ per mL, which was then cultured at 37 °C for 5–7 days. Both cell and virus controls were also set up as a comparison. Subsequently, the inhibition of virus infection to the cells was observed, and the neutralizing antibody titer against the live SARS-CoV-2 virus was measured as the reciprocal of the serum dilution for 50% neutralization of viral infection.

### Live SARS-CoV-2 challenge experiment

The mouse challenge experiments were performed in the BSL3 facility of the National Institute for Viral Disease Control and Prevention, Chinese Center for Disease Control and Prevention (China CDC), Beijing, China. A total of 40 female hACE2 B6-Tg transgenic mice (purchased from Beijing Huafukang Bioscience Co., Ltd, China.), aged 8–10 weeks and weighted 16–25 g, were used for the live SARS-CoV-2 challenge experiment, which were randomly divided into eight groups (5 mice per group): three vaccine groups, three saline groups and two control groups. The mice in vaccine groups were immunized intramuscularly with two doses (2 µg/dose) of mutI-tri-RBD vaccine candidate on Day 0 and Day 21. In the saline and control groups, the mice were intramuscularly injected with physiological saline on Day 0 and Day 21. On day 7 after the second vaccination, the blood from the caudal vein of the immunized mice was collected, and serum was separated to measure the titer of neutralizing antibodies using a pseudo-virus neutralization assay. On day 14 after the second immunization, two vaccine groups, as well as corresponding two saline groups, were challenged with the prototype and Beta live SARS-CoV-2 viruses, respectively, and a control group was challenged with saline as a blank control. On day 19 after the whole immunization, the remaining one vaccine group and one saline group were challenged with the Delta live virus, and correspondingly a control group was challenged with saline as a blank control. In the challenge experiments, each animal was received 1.5 × 10^5^ TCID_50_ of live virus or 15 μL saline via the intranasal route. During the challenge experiment, the changes in the body weight of the mice were measured, and the living status of the mice, including survival or not, muscle strength, vitality, and so on, were observed and recorded. Five days after the prototype and Beta virus challenges, or six days after the Delta virus challenge, all mice were euthanized and the lung tissues were collected. The viral RNA was extracted from lung tissues by using a nucleic acid extraction kit (QIAGEN, No. 52906), and the viral load was evaluated by using a 2019-nCoV (N, ORF1ab, and S genes) nucleic acid detection kit (PCR-fluorescence probing). In addition, the histopathological sections of the lung tissues of the mice were stained with hematoxylin and eosin (H&E), and a pathological examination was performed.

### Quantification and statistical analysis

One-way analysis of variance with Sidak’s multiple comparison test was used to determine the statistical significance of the difference between multiple groups, and Student’s *t-*test was used for statistical analysis between two groups. *P < 0.05, **P < 0.01, ***P < 0.001, ****P < 0.0001, ns, not significant. Details can be found in the figure legend.

## Supplementary information

Supplementary Information

Video S1

Video S2

## Data Availability

All data supporting the findings of this study are available within the paper or the Supplemental Information. Any additional information required to reanalyze the data reported in this paper is available from the corresponding author Q. M. L. (liqiming189@163.com) upon request.
