# Supplementary material for: Design of a mutation-integrated trimeric RBD with broad protection against SARS-CoV-2
Source: Cell Discov. 2022 Feb 15;8:17. doi: 10.1038/s41421-022-00383-5 (PMC8847466; doi:10.1038/s41421-022-00383-5)
Supplement: Supplementary file 1 — Supplementary Information [file 41421_2022_383_MOESM1_ESM.pdf]

Supplementary Information

Table S1 RBD-specific IgG titers against the prototype SARS-CoV-2 strain for each mouse induced by mutI-tri-RBD with different immunization regimens and doses.

Shots	Doses	Mouse1	Mouse2	Mouse3	Mouse4	Mouse5	Mouse6	Mouse7	Mouse8	GMT
2 shots	0.125μg	80000	40000	80000	80000	160000	80000	80000	40000	73360
	0.5μg	160000	80000	160000	160000	640000	80000	80000	160000	146721
	2.0μg	40000	80000	80000	320000	80000	160000	25*	80000	97521
3 shots	0.125μg	320000	640000	320000	320000	320000	320000	320000	640000	380546
	0.5μg	640000	320000	640000	640000	640000	640000	640000	640000	586883
	2.0μg	320000	20000	160000	640000	640000	640000	640000	320000	293441
Control		25	25	25	25	25	25	25	25	25

*This vaccinated mouse displayed negative IgG, which was excluded from the GMT calculation and statistical analysis.

Table S2 RBD-specific IgG titers against the prototype SARS-CoV-2 strain for each mouse induced by mutI-tri-RBD compared with those induced by homo-tri-RBD.

Vaccine	Shots	Doses	Mouse1	Mouse2	Mouse3	Mouse4	Mouse5	Mouse6	Mouse7	Mouse8	GMT
mutI-tri-RBD	2 shots	2.0μg	40000	80000	80000	320000	80000	160000	25*	80000	97521
	3 shots	0.5μg	640000	320000	640000	640000	640000	640000	640000	640000	586883
Homo-tri-RBD	2 shots	2.0μg	160000	320000	20000	160000	320000	80000			126992
	3 shots	0.5μg	320000	160000	320000	320000	640000	320000			320000
Control			25	25	25	25	25	25	25	25	25

*data excluded from the GMT calculation and statistical analysis.

Table S3 Pseudo-virus neutralizing antibody titers against the prototype SARS-CoV-2 strain for each mouse induced by mutI-tri-RBD with different immunization regimens and doses.

Shots	Doses	Mouse1	Mouse2	Mouse3	Mouse4	Mouse5	Mouse6	Mouse7	Mouse8	GMT
2 shots	0.125μg	623	169	361	724	3759	703	439	488	594
	0.5μg	877	633	1039	1172	902	1071	850	673	884
	2.0μg	1181	1725	861	5014	2066	694	20*	1901	1574
3 shots	0.125μg	12438	40498	10042	12707	14350	34440	9497	19254	16615
	0.5μg	13276	15193	55004	83503	24670	45870	28825	41261	32507
	2.0μg	8547	455	1813	35464	18675	39100	29746	15078	9753
Control		20	20	20	20	20	20	20	20	20

*data excluded from the GMT calculation and statistical analysis.

Table S4 Pseudo-virus neutralizing antibody titers against the prototype SARS-CoV-2 strain for each mouse induced by mutI-tri-RBD compared with those induced by homo-tri-RBD.

Vaccine	Shots	Doses	Mouse1	Mouse2	Mouse3	Mouse4	Mouse5	Mouse6	Mouse7	Mouse8	GMT
mutI-tri-RBD	2 shots	2.0μg	1181	1725	861	5014	2066	694	20*	1901	1574
	3 shots	0.5μg	13276	15193	55004	83503	24670	45870	28825	41261	32507
Homo-tri-RBD	2 shots	2.0μg	2047	6116	206	5303	2500	6505			2461
	3 shots	0.5μg	12257	2818	36369	33299	34191	18201			17216
Control			20	20	20	20	20	20	20	20	20

*data excluded from the GMT calculation and statistical analysis.

Table S5 Live virus neutralizing antibody titers against the prototype SARS-CoV-2 strain for each mouse induced by mutI-tri-RBD with different immunization regimens and doses.

Shots	Doses	Mouse1	Mouse2	Mouse3	Mouse4	Mouse5	Mouse6	Mouse7	Mouse8	GMT
2 shots	0.125μg	640	40	160	640	1280	320	160	320	293
	0.5μg	640	320	320	320	320	320	320	320	349
	2.0μg	640	1280	320	1280	1280	320	20*	320	640
3 shots	0.125μg	5120	20480	5120	2560	5120	5120	1280	5120	4695
	0.5μg	10240	5120	10240	20480	10240	20480	10240	20480	12177
	2.0μg	2560	320	640	20480	5120	10240	20480	5120	3948
Control		20	20	20	20	20	20	20	20	20

*data excluded from the GMT calculation and statistical analysis.

Table S6 Live virus neutralizing antibody titers against the prototype SARS-CoV-2 strain for each mouse induced by mutI-tri-RBD compared with those induced by homo-tri-RBD.

Vaccine	Shots	Doses	Mouse1	Mouse2	Mouse3	Mouse4	Mouse5	Mouse6	Mouse7	Mouse8	GMT
mutI-tri-RBD	2 shots	2.0μg	640	1280	320	1280	1280	320	20*	320	640
	3 shots	0.5μg	10240	5120	10240	20480	10240	20480	10240	20480	12177
Homo-tri-RBD	2 shots	2.0μg	1280	1280	20	1280	1280	5120			806
	3 shots	0.5μg	5120	640	20480	5120	10240	2560			4561
Control			20	20	20	20	20	20	20	20	20

*data excluded from the GMT calculation and statistical analysis.

Table S7 Pseudo-virus neutralizing antibody titers against the Delta (B.1.617.2) SARS-CoV-2 strain for each mouse induced by mutI-tri-RBD compared with those induced by homo-tri-RBD.

Vaccine	Shots	Doses	Mouse1	Mouse2	Mouse3	Mouse4	Mouse5	Mouse6	Mouse7	Mouse8	GMT
mutI-tri-RBD	2 shots	2.0μg	302	677	1057	2075	316	435	20*	853	657
	3 shots	0.5μg	4812	11721	10969	10816	7415	13878	13990	26200	11227
Homo-tri-RBD	2 shots	2.0μg	229	2096	20	1152	100	100			219
	3 shots	0.5μg	1448	1963	8323	2805	1671	3480			2698
Control			20	20	20	20	20	20	20	20	20

*data excluded from the GMT calculation and statistical analysis.

Table S8 Live virus neutralizing antibody titers against the Delta (B.1.617.2) SARS-CoV-2 strain for each mouse induced by mutI-tri-RBD compared with those induced by homo-tri-RBD.

Vaccine	Shots	Doses	Mouse1	Mouse2	Mouse3	Mouse4	Mouse5	Mouse6	Mouse7	Mouse8	GMT
mutI-tri-RBD	2 shots	2.0μg	80	80	320	640	80	320	20*	80	160
	3 shots	0.5μg	2560	5120	10240	5120	5120	5120	5120	5120	5120
Homo-tri-RBD	2 shots	2.0μg	40	320	20	320	20	20			57
	3 shots	0.5μg	1280	160	1280	1280	640	1280			806
Control			20	20	20	20	20	20	20	20	20

*data excluded from the GMT calculation and statistical analysis.

Table S9 Pseudo-virus neutralizing antibody titers against the Beta (B.1.351) SARS-CoV-2 strain for each mouse induced by mutI-tri-RBD compared with those induced by homo-tri-RBD.

Vaccine	Shots	Doses	Mouse1	Mouse2	Mouse3	Mouse4	Mouse5	Mouse6	Mouse7	Mouse8	GMT
mutI-tri-RBD	2 shots	2.0μg	1567	2456	2197	5000	2589	661	20*	2646	2118
	3 shots	0.5μg	25000	18374	25000	25000	25000	25000	25000	25000	24056
Homo-tri-RBD	2 shots	2.0μg	872	431	68	1253	587	3164			626
	3 shots	0.5μg	13688	1574	25000	22035	16778	2508			8907
Control			20	20	20	20	20	20	20	20	20

*data excluded from the GMT calculation and statistical analysis.

Table S10 Live virus neutralizing antibody titers against the Beta (B.1.351) SARS-CoV-2 strain for each mouse induced by mutI-tri-RBD compared with those induced by homo-tri-RBD.

Vaccine	Shots	Doses	Mouse1	Mouse2	Mouse3	Mouse4	Mouse5	Mouse6	Mouse7	Mouse8	GMT
mutI-tri-RBD	2 shots	2.0μg	1280	1280	1280	2560	5120	640	20*	2560	1723
	3 shots	0.5μg	20480	20480	10240	20480	10240	20480	20480	20480	17222
Homo-tri-RBD	2 shots	2.0μg	1280	320	160	1280	320	1280			570
	3 shots	0.5μg	5120	640	10240	5120	5120	1280			3225
Control			20	20	20	20	20	20	20	20	20

*data excluded from the GMT calculation and statistical analysis.

Table S11 Neutralizing antibody responses against 23 various SARS-CoV-2 pseudo-virus strains for each mouse elicited by mutI-tri-RBD compared with those induced by homo-tri-RBD.

Pseudo-viruses	Vaccine	Mouse1	Mouse2	Mouse3	Mouse4	Mouse5	Mouse6	Mouse7	Mouse8	GMT
prototype	mutI-tri-RBD	3231	4748	8538	20*	1767	3318	7724	3587	4152
	Homo-tri-RBD	6927	4296	3465	4160	204	7823			2970
	Control	20	20	20	20	20	20	20	20	20
D614G	mutI-tri-RBD	2313	3478	8025	20*	2041	1223	1192	5163	2680
	Homo-tri-RBD	8657	4362	1535	10626	235	3702			2851
	Control	20	20	20	20	20	20	20	20	20
Delta (B.1.617.2)	mutI-tri-RBD	836	845	8382	20*	2729	682	2739	1935	1788
	Homo-tri-RBD	270	426	515	3338	20	4527			512
	Control	20	20	20	20	20	20	20	20	20
Beta (B.1.351)	mutI-tri-RBD	1662	6079	18499	20*	4872	2987	5603	8531	5377
	Homo-tri-RBD	3597	2128	2413	2936	130	931			1369
	Control	20	20	20	20	20	20	20	20	20
501Y.V2-1	mutI-tri-RBD	2400	3234	13903	20*	4716	2441	2218	5076	3911
	Homo-tri-RBD	8911	1754	1758	2004	116	620			1259
	Control	20	20	20	20	20	20	20	20	20
501Y.V2-3	mutI-tri-RBD	2808	3209	17427	20*	5742	2871	3191	5499	4627
	Homo-tri-RBD	9470	5645	1917	2383	116	2133			1980
	Control	20	20	20	20	20	20	20	20	20
Alpha (B.1.1.7)	mutI-tri-RBD	875	1023	6096	20*	1206	806	953	921	1246
	Homo-tri-RBD	4352	2738	987	3903	58	4857			1532
	Control	20	20	20	20	20	20	20	20	20
Gamma (P.1)	mutI-tri-RBD	1828	8697	9013	20*	9787	488	9245	6347	4546
	Homo-tri-RBD	16656	5388	2731	3358	163	1620			2451
	Control	20	20	20	20	20	20	20	20	20
Zeta (P.2)	mutI-tri-RBD	978	1340	7934	20*	2417	2717	1986	5137	2548
	Homo-tri-RBD	2152	937	1441	830	74	323			622
	Control	20	20	20	20	20	20	20	20	20
Epsilon (B.1.429)	mutI-tri-RBD	1591	3156	9059	20*	3864	1161	4857	5173	3388
	Homo-tri-RBD	3207	619	1168	7624	84	1612			1157
	Control	20	20	20	20	20	20	20	20	20
Eta (B.1.525)	mutI-tri-RBD	2145	4040	18056	20*	5031	4213	2100	2376	4006
	Homo-tri-RBD	2347	935	2033	1201	110	340			764
	Control	20	20	20	20	20	20	20	20	20
Iota (B.1.526)	mutI-tri-RBD	1549	1772	11990	20*	2813	1593	2326	2248	2585
	Homo-tri-RBD	3353	803	1471	962	99	231			666
	Control	20	20	20	20	20	20	20	20	20
D614G+K417N	mutI-tri-RBD	1471	1374	14150	20*	2717	2791	3068	6386	3299
	Homo-tri-RBD	15025	5965	3318	52006	248	2867			4715
	Control	20	20	20	20	20	20	20	20	20

D614G+E484K	mutI-tri-RBD	1754	3347	8443	20*	3750	2491	2664	2714	3188
	Homo-tri-RBD	7645	1264	2493	1674	224	727			1368
	Control	20	20	20	20	20	20	20	20	20
D614G+N501Y	mutI-tri-RBD	1061	931	5579	20*	1099	584	992	1819	1303
	Homo-tri-RBD	5419	1069	1193	6153	90	3061			1506
	Control	20	20	20	20	20	20	20	20	20
D614G+L452R	mutI-tri-RBD	700	1348	6962	20*	2763	1044	3043	2080	1982
	Homo-tri-RBD	1975	187	1043	3567	127	1894			832
	Control	20	20	20	20	20	20	20	20	20
D614G+E484Q	mutI-tri-RBD	1934	2639	10229	20*	3384	2003	1772	5194	3176
	Homo-tri-RBD	2937	1457	1605	1807	144	508			984
	Control	20	20	20	20	20	20	20	20	20
D614G+T478K	mutI-tri-RBD	3160	1712	9771	20*	2475	1055	1294	2151	2340
	Homo-tri-RBD	3732	1827	681	10667	57	28227			2074
	Control	20	20	20	20	20	20	20	20	20
D614G+K417N +N501Y	mutI-tri-RBD	1639	4371	12227	20*	2898	3503	2116	5297	3726
	Homo-tri-RBD	13824	5277	4108	18796	228	3664			4092
	Control	20	20	20	20	20	20	20	20	20
D614G+E484K +N501Y	mutI-tri-RBD	2127	3833	6309	20*	3315	2782	1714	4544	3233
	Homo-tri-RBD	4904	797	1736	1402	104	446			872
	Control	20	20	20	20	20	20	20	20	20
B.1.1.7+E484K	mutI-tri-RBD	1301	1728	7457	20*	2271	2286	2586	2968	2533
	Homo-tri-RBD	1642	850	982	1083	61	376			569
	Control	20	20	20	20	20	20	20	20	20
D614G+K417N +E484K	mutI-tri-RBD	2652	5019	19271	20*	5507	3297	4385	6404	5382
	Homo-tri-RBD	7236	3412	2777	2163	81	839			1468
	Control	20	20	20	20	20	20	20	20	20
D614G+K417N +E484K+N501Y	mutI-tri-RBD	3281	5195	13638	20*	5160	4661	5603	5627	5616
	Homo-tri-RBD	9101	2128	2413	2936	205	1217			1803
	Control	20	20	20	20	20	20	20	20	20

*data excluded from the GMT calculation and statistical analysis.

Table S12 Pseudo-virus neutralizing antibody titers against the prototype, Delta (B.1.617.2) and Beta (B.1.351) SARS-CoV-2 strains, respectively, induced by mutI-tri-RBD for each hACE2 transgenic mouse in vaccine groups compared with those in saline groups.

Group	Mouse1	Mouse2	Mouse3	Mouse4	Mouse5	GMT
Vaccine group against prototype	864	293	336	3921	14535	1371
Saline group against prototype	20	20	20	20	20	20
Vaccine group against Delta	1658	1572	946	5505	2328	1995
Saline group against Delta	20	20	20	20	20	20
Vaccine group against Beta	723	1071	2265	7930	1156	1743
Saline group against Beta	20	20	20	20	20	20

Table S13 Body weight changes of the hACE2 transgenic mouse in each group during live virus challenge experiments.

Group	Mouse	Body weight (g)						
		Day0	Day1	Day2	Day3	Day4	Day5	Day6
Vaccine group against prototype	1#	39.90	39.30	39.60	/*	37.70	38.67	-**
	2#	34.07	35.10	35.20	/	35.10	35.63	-
	3#	35.10	35.00	35.60	/	34.65	35.77	-
	4#	35.13	36.90	37.27	/	36.20	37.03	-
	5#	37.10	38.13	38.67	/	39.10	39.63	-
Vaccine group against Delta	1#	33.10	32.70	32.10	33.30	35.80	37.40	38.60
	2#	31.13	31.30	31.20	30.50	30.83	30.87	31.07
	3#	30.40	31.90	33.00	33.00	33.40	34.80	34.93
	4#	35.33	36.03	35.90	35.30	35.80	36.40	36.57
	5#	34.40	36.30	36.80	37.40	37.63	37.60	37.90
Vaccine group against Beta	1#	34.77	36.70	36.80	/	33.40	38.27	-
	2#	39.00	38.47	38.50	/	37.50	38.73	-
	3#	32.40	31.67	31.80	/	29.13	31.03	-
	4#	31.00	30.90	31.00	/	31.40	31.37	-
	5#	32.07	32.17	32.50	/	37.60	33.33	-
Saline group against prototype	1#	35.70	35.73	35.37	/	35.40	34.60	-
	2#	37.77	38.33	38.00	/	38.40	37.20	-
	3#	51.63	51.40	49.80	/	50.40	49.33	-
	4#	32.37	32.03	31.67	/	32.30	31.20	-
	5#	32.30	32.83	32.40	/	33.60	32.13	-
Saline group against Delta	1#	33.00	32.60	32.60	32.80	33.17	32.77	31.70
	2#	34.40	34.90	34.80	34.80	35.70	35.70	34.83
	3#	33.70	33.50	33.60	33.80	33.90	33.70	30.40
	4#	31.67	31.47	31.80	32.70	32.70	32.90	32.13
	5#	33.53	33.80	34.20	34.40	34.70	36.60	34.07
Saline group against Beta	1#	34.90	33.87	33.60	/	34.90	33.20	-
	2#	39.33	38.43	37.67	/	37.70	36.30	-
	3#	31.57	30.63	30.27	/	28.03	died	-

	4#	35.47	35.90	35.30	/	34.90	32.43	-
	5#	38.17	38.20	37.60	/	37.90	36.40	-
Control group 1	1#	35.13	34.73	34.83	/	34.60	34.90	-
	2#	33.93	34.20	34.53	/	33.50	33.80	-
	3#	36.00	36.47	37.03	/	37.50	37.47	-
	4#	34.37	35.43	36.13	/	35.77	36.40	-
	5#	34.37	34.13	34.70	/	34.20	34.47	-
Control group 2	1#	32.13	32.90	32.80	32.90	33.47	32.90	33.10
	2#	34.20	34.70	35.00	35.10	34.97	35.20	35.67
	3#	35.33	35.93	36.10	36.30	36.80	36.70	37.00
	4#	35.10	35.57	35.80	36.90	37.87	38.00	38.50
	5#	37.10	38.90	38.40	38.40	39.00	39.67	40.40

* “/” means data not recorded. ** “-” represents end of challenge experiment.

Table S14 Viral RNA loads monitored by using a 2019-nCoV (N, ORF1ab and S genes) nucleic acid detection kit in the lung tissue of each mouse in live virus challenge experiments.

Group	Viral RNA Gene	Viral RNA loads (copies/ml)				
		Mouse 1	Mouse 2	Mouse 3	Mouse 4	Mouse 5
Vaccine group against prototype	N	—*	—	—	—	—
	ORF 1ab	—	—	—	—	—
	S	—	—	—	—	19833
Vaccine group against Delta	N	—	—	—	—	—
	ORF 1ab	—	—	—	—	—
	S	1882	—	842	—	—
Vaccine group against Beta	N	—	—	—	—	—
	ORF 1ab	—	—	—	—	—
	S	—	—	—	—	—
Saline group against prototype	N	1729887	1676657	1801328	1218373	3361367
	ORF 1ab	874126	933918	1042956	733974	2460685
	S	2198108	2185097	2589978	2043712	5080851
Saline group against Delta	N	667480	1140631	632226	789538	447930
	ORF 1ab	436535	645305	353303	477782	263199
	S	1004926	1409056	873287	1080953	676802
Saline group against Beta	N	2169874	2804585	died	2131728	1468370
	ORF 1ab	1396709	1471441		1405689	720513
	S	2986969	3433655		3001863	1847025
Control group 1	N	—	—	—	—	—
	ORF 1ab	—	—	—	—	—
	S	—	1358	—	4966	—
Control group2	N	—	—	—	—	—
	ORF 1ab	—	—	—	—	—
	S	1100	—	—	—	—

*“-” means viral load less than the detectable limit (< 500 copies/ml).

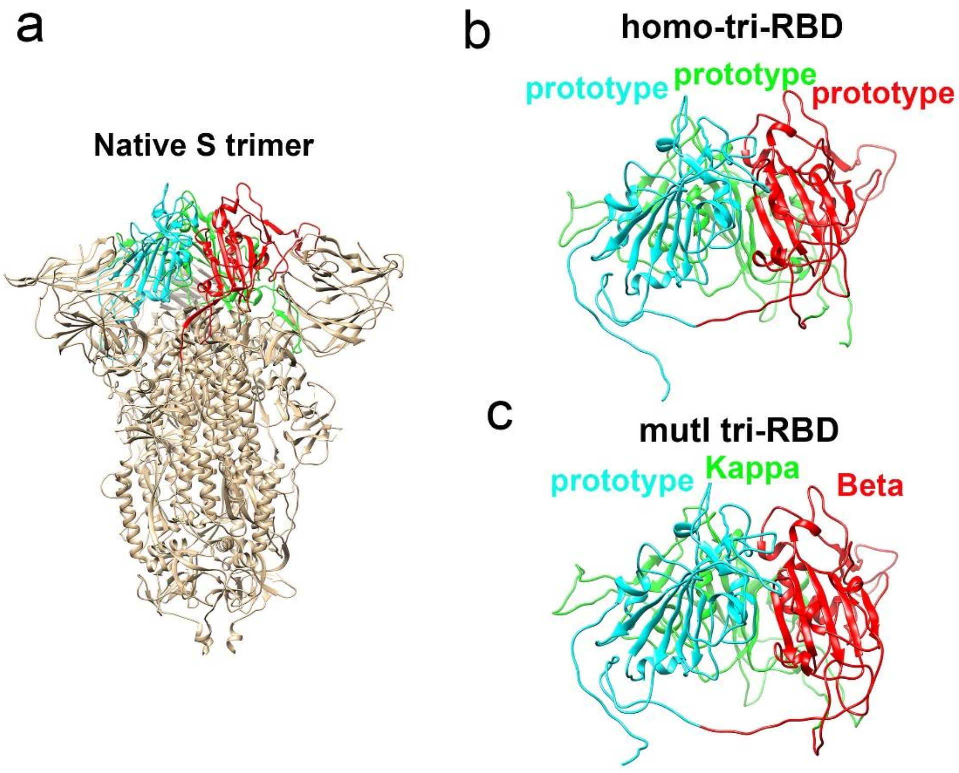

Fig. S1: Structural modelling and molecular dynamics (MD) simulation of the designed mutI-tri-RBD and homo-tri-RBD. a Native structure of S protein trimer. Cyan, red and green colors represent three RBDs which were assembled into a trimeric arrangement. **b** Structural modelling of the homo-tri-RBD protein by Modeller software using the native structure of S protein as the template. **c** Structural modelling of the mutI-tri-RBD protein by Modeller software using the native structure of S protein as the template.

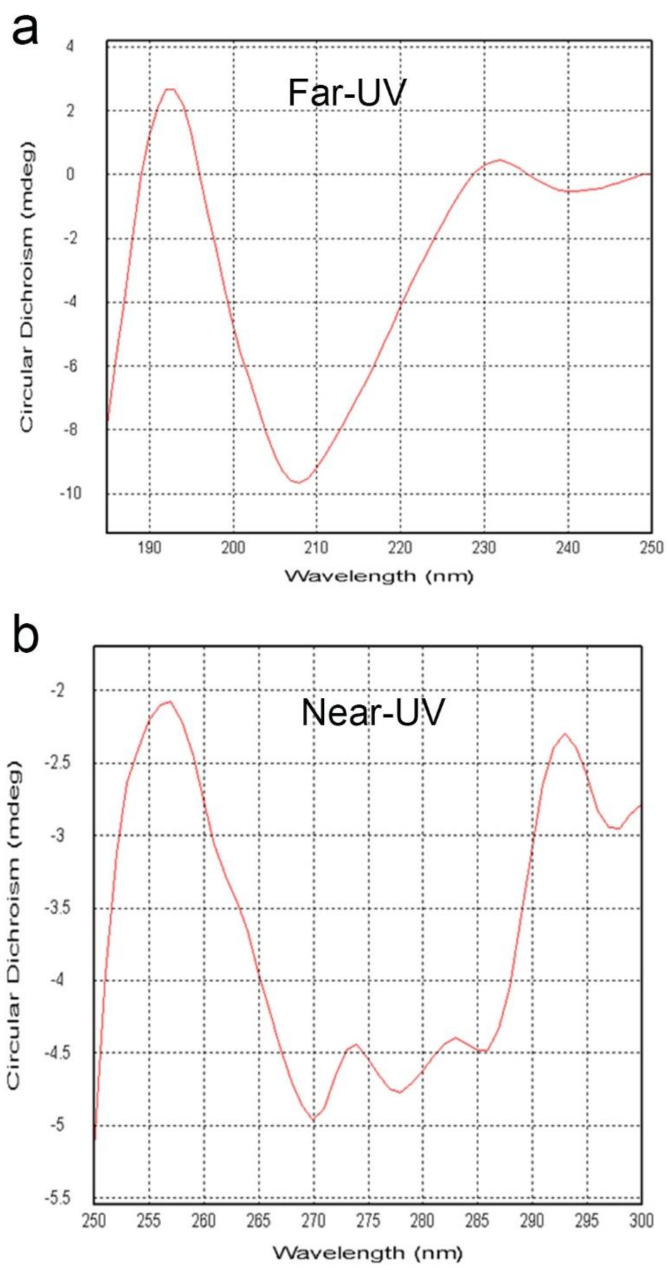

Fig. S2: Far-ultraviolet (UV) and near-UV spectra acquired by Circular Dichroism (CD) to estimate the content of different secondary structures in the recombinant mutI-tri-RBD.

a Far-UV spectra acquired by CD. **b** Near-UV spectra obtained by CD.

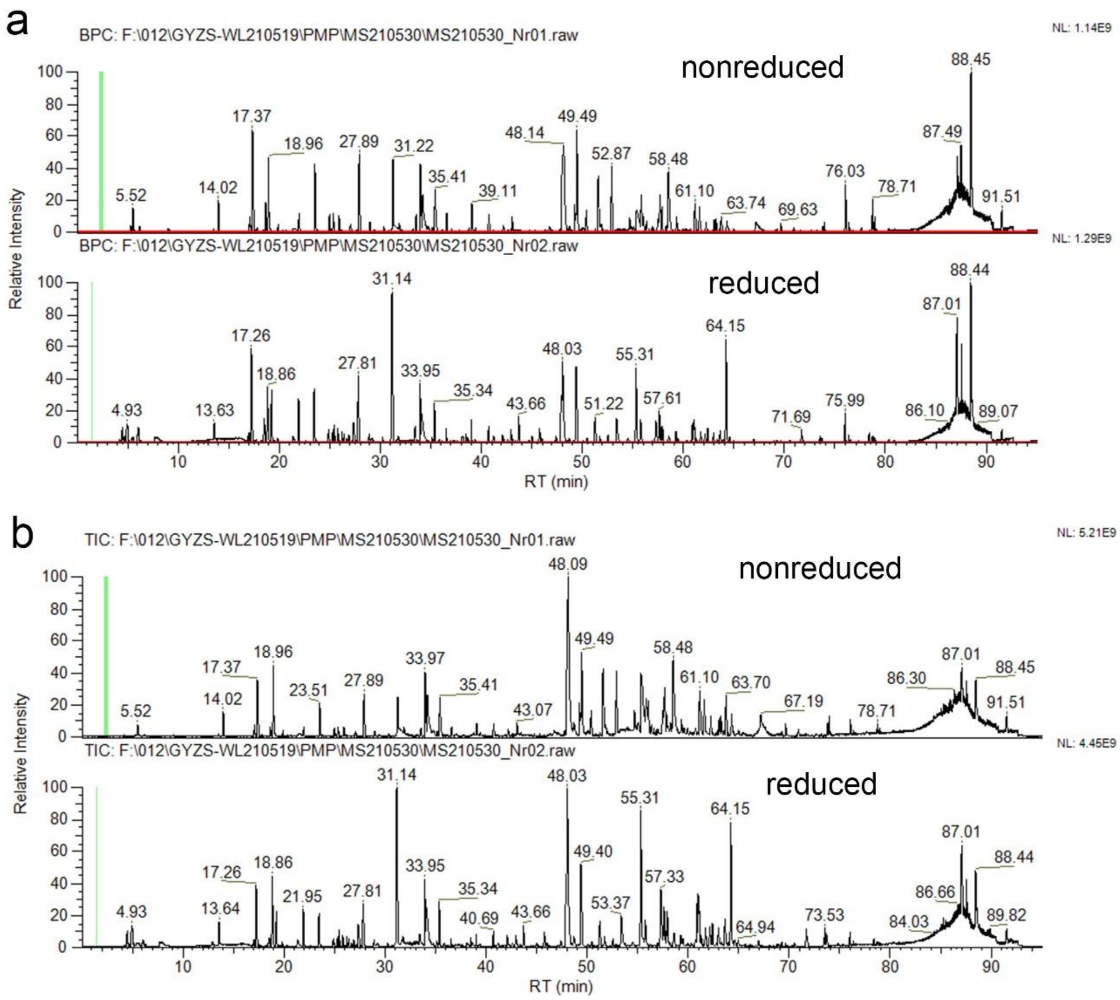

Fig. S3: Base Peak Chromatogram (BPC) and Total Ion Chromatogram (TIC) to identify the disulfide-bonds in mutI-tri-RBD by UPLC-MS. a BPC for the nonreduced and reduced protein sample. **b** TIC for the nonreduced and reduced protein sample.

Video S1 Molecular dynamics simulation of mutI-tri-RBD.

Video S2 Molecular dynamics simulation of homo-tri-RBD.
